# A detailed analysis of the Czernik 38 cluster and its associated tidal tail, utilizing Gaia DR3 and 2MASS

**DOI:** 10.1038/s41598-025-32463-3

**Published:** 2026-02-12

**Authors:** Nasser M. Ahmed

**Affiliations:** National Research Institute of Astronomy and Geophysics (NRIAG), 11421 Helwan, Cairo Egypt

**Keywords:** Star cluster, Gaia DR3, CMD, Parallax, Proper motion, Distance, Membership, Mass function, Tidal tail, Binary cluster, Astronomy and planetary science, Mathematics and computing, Optics and photonics, Physics

## Abstract

This study provides a thorough investigation of the open cluster Czernik 38, employing photometric and astrometric data from Gaia DR3 and 2MASS. Our analysis refines the fundamental parameters of the cluster, including its structure, kinematics, evolutionary status, age, and morphology. To evaluate membership, we utilized the pyUPMASK Python package in conjunction with the HDBSCAN algorithm. The main focus of this research is our novel method of assigning a membership probability at each radius, instead of using a singular value for the entire cluster. One of the main outcomes of our research indicates that there is an elongated structure and a leading tidal tail that aligns with the orbital trajectory of the cluster. This tidal phenomenon arises due to orbital differential rotation. Furthermore, we have discovered a new star cluster located 32 arcmin from the center of Czernik. This cluster may serve as a companion to the Czernik 38 cluster in a binary cluster system or a complex colliding system; we will explore this further in subsequent research. According to Gaia, the distance modulus of the cluster and the color excess $E(G_{BP} - G_{RP})$ are measured at 12.69$\pm$ 0.08 mag and 2.40 $\pm$ 0.04 mas, respectively. Additionally, from 2Mass, the distance modulus is 12.87 $\pm$ 0.93 mag, while the color excess $E(J-K_s)$ is 0.89 $\pm$ 0.2 mag. Moreover, the cluster age is determined to be 115.0$\pm$ 20.3 Myr. The components of proper motion ($\mu _{\alpha } \cos \delta$, $\mu _{\delta }$) and the parallax ($\varpi$) are found as -2.41 $\pm$ 0.328 mas yr$^{-1}$, -5.263 $\pm$ 1.063 mas yr$^{-1}$, and 0.21 $\pm$ 0.083 mas, respectively. The calculated mean Gaia distances are roughly 3580.4 $\pm$ 230.5 pc, which is in agreement with the photometric data from the Gaia and 2Mass data, within the error. There are 37 stars that exhibit radial velocity with average 46.1 $\pm$ 8.54$km s^{-1}$, which allows us to derive orbital parameters using the galpy python package. As a result, the cluster is moving parallel to the Galactic plane towards the Galactic center. We have identified a novel category of pre-main sequence stars that form a distinct branch in the right of Color-Magnitude Diagram (CMD). These stars exhibit lower temperatures and surface gravity compared to main sequence stars. This implies that there exists a significant rate of star formation within the Czernik 38 cluster. Furthermore, we have discovered many faint blue stars in Czernik 38, as well as in the newly identified clusters, which could potentially be white dwarf stars.

## Introduction

Open clusters (OCs) are significant in improving our insight into stellar evolution and the structure of galaxies. These gravitationally bound groups of stars, which share similar ages and chemical abundances, are crucial for elucidating the formation history of the disc of the Milky Way. They are formed from stars that developed under almost identical physical conditions and within a short time frame, thereby acting as exceptional indicators of the physical and dynamic states of the interstellar medium. They usually consist of several dozen to thousands of stars situated at comparable distances. OCs are particularly useful for studying the structure, kinematics, and various properties of the Milky Way^1–4^, e.g..

**Fig. 1:**
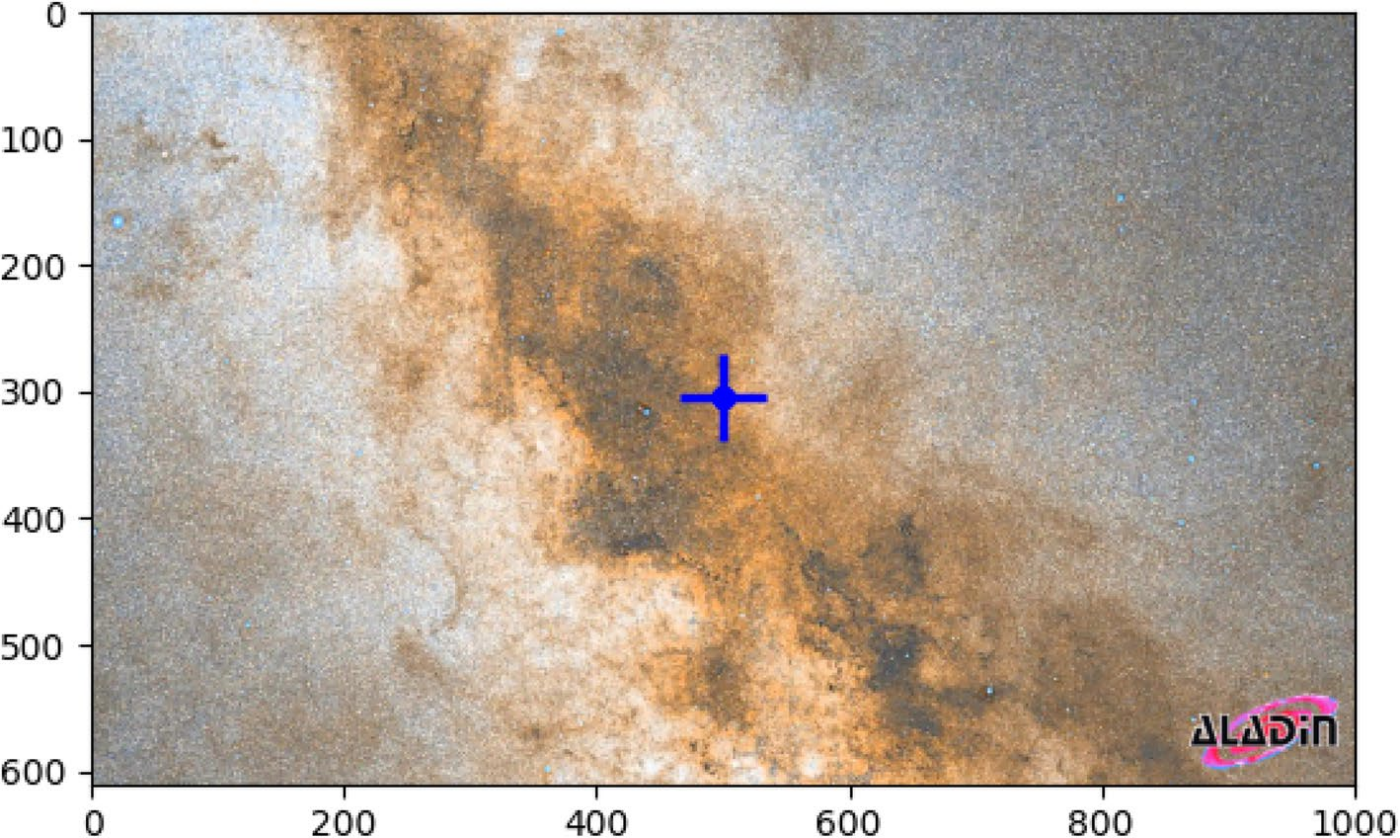
The Czernik 38 cluster’s position (blue “+” symbol) is superimposed on the AlaDin (PanSTARS^27^) footnotehttps://aladin.cds.unistra.fr/aladin.gml image depicting the Carina-Sagittarius spiral arm. Take note of the dim area, which is composed of the dust and dense gaseous.

Czernik 38 is a rich open star cluster and it is discovered by^5^. It resides in the direction of the boundary of the Carina-Sagittarius spiral arms, directed toward the Scutum-Crux arm, with coordinates $\alpha$ = 18$^h$ 49$^m$ 48$^s$ and $\delta$ = +4$^\circ$ 58’ 10” (J2000.0), which corresponds to Galactic coordinates of $l = 37.17^\circ$ and $b = 2.62^\circ$ (located in the first Galactic quadrant. It is located in the area of the high stellar-density background; see Fig. 1. A significant characteristic of it is a considerable reddening, which suggests that it is positioned within or behind the Sagittarius arm. Therefore, this imposes a constraint on the determination of the distance.

Despite the significant position and richness of Czerink 38, there exists a litel regarding investigations of it. According to^6^, the only parameter noted is the cluster’s angular diameter of 10’^7^. suggested that the cluster is at least 1 Gyr old and at a heliocentric distance of 1.28 Kpc. Furthermore, he has determined that the color excess E(B-V) is 1.09±0.02 mag. While^8^ has determined that color excess E(B-V), the age and distance are 1.25 ± 0.10 mag, 600 Myr and 1.9 Kpc, respectively.

One of the significant characteristics of OCs is the existence of tidal tails. These tidal tails are stellar structures that extend from the main body of the cluster, arising from the gravitational interactions between the cluster and the Galactic potential or a giant molecular cloud^9^. In young OCs (ages $\lesssim$ 100 Myr), these structures could be the result of the remnants of the giant molecular clouds (GMCs) from which the clusters developed or due to rapid gas expulsion^10,11^, e.g.,. In older clusters (ages $\gtrsim$ 100 Myr), mechanisms such as two-body relaxation or external forces, including disk shocks, are likely responsible for the stripped stars observed in tidal tails^12^. Several young OCs have been documented with tail-like formations, including IC 2391, IC 2602, NGC 2451 A, and NGC 2547^10^. Furthermore, a growing number of older OCs have been identified with tidal tails, such as Hyades^13–15^ and NGC 6774^11^. Recently^16^, conducted a study on tidal tails using Gaia EDR3 data and discovered 72 OCs exhibiting tidal tails; however, Czerink 38 was absent from their findings related to tidal tails.

Numerous techniques exist in the literature for identifying the tidal tail in OCs, including the work of^17^, who employed a machine-learning algorithm to detect an extended structure beyond the tidal radius. Moreover^18^, has developed an innovative technique for tracking escape stars originating from nearby clusters, utilizing the radial velocity data provided by Gaia. Others notable attempts to detect tidal tails of OCs; NGC 725^17,19^, IC 4756^20^, Ruprecht 147^21^ NGC 2506^22^, NGC 2516/NGC 6633^23^ and Alpha Persi^24^.

Most of these methods have identified S-shaped structures, which are characterized by leading and trailing tails. However, in this study, we have observed the tidal tail as only a leading tail in front of the cluster and aligned with the direction of motion. Additionally, in our previous investigations of the King 13 cluster^25^, we reported similar findings. We have implemented a novel technique for identifying cluster members by integrating the pyUpmask probability with the King model.

Gaia DR3’s data presents outstanding accuracy in 5D astrometry, including the evaluation of positions, proper motions, and parallax, as well as thorough photometric measurements. The present study explores the key astrophysical features of Czernik 38, making use of the astrometric and photometric data available from Gaia DR3^26^. In addition, we analyze the orbital parameters and kinematic characteristics of this system. Furthermore, the cluster’s morphology is examined, with a focus on identifying any features related to tidal tails.

Furthermore, we investigate two critical issues that significantly influence the research in the field of open star cluster studies. The first topic addresses the limitations of data (Section "The data limits"), and the second topic concerns the cutoff value for membership probability (Section "The probability cut-off value").

The paper is organized as follows. Section “Data” outlines the criteria used to extract the initial data sample from Gaia DR3. The cluster’s structure, along with its radial density profile, is described in Section "Radial density profile and cluster structure". In Section “Membership determination”, we present the astrometric analysis, cluster membership determination, and the identification of the cluster’s center. The photometric properties of the cluster members, as well as the detection of the tidal tail, are discussed in Sections "The photometry of Czernik 38 cluster". The cluster kinematics and dynamics analysis will be in Section "The cluster dynamics and kinematics". The details of tidal tail in Czerink 38 cluster will be given in Section "The morphology and the tidal tail of Czernik 38". Finally, the main conclusions are summarized in Section "Summary and conclusions".

## Data

In this study, we utilize two comprehensive and complementary datasets, Gaia DR3 and 2MASS, to examine the open cluster Czernik 38. These datasets provide a robust foundation for identifying cluster members, extracting astrophysical parameters, and examining the structure and kinematics of the cluster. Below, we present a summary of the essential characteristics and significance of these datasets in relation to the current research.

### Gaia DR3 data

**Fig. 2:**
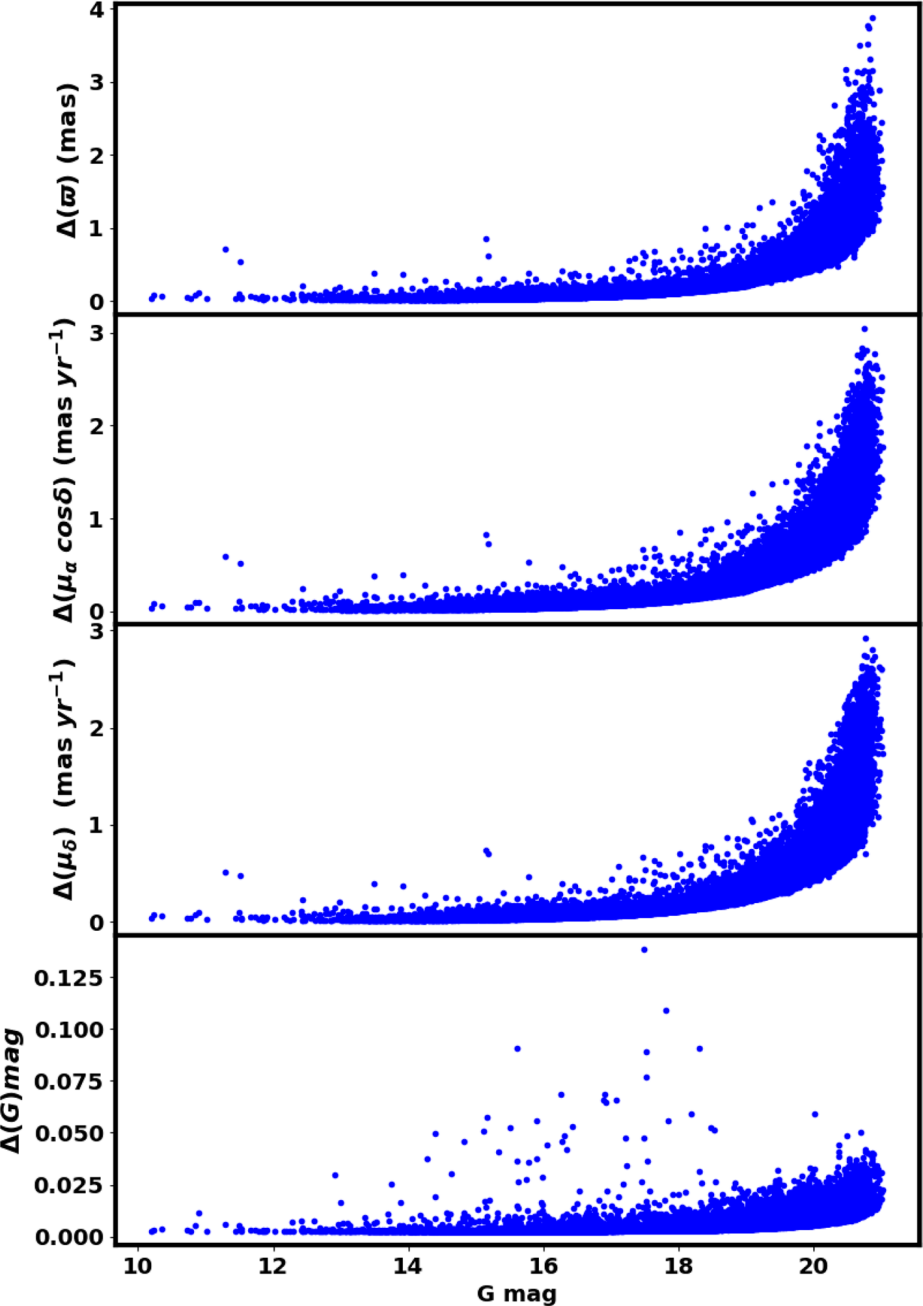
The graph depicting G magnitude in relation to the errors of parallax, proper motion, and G magnitude.

We retrieved data for Czernik 38 from the Gaia DR3 catalog^26^. The dataset comprises proper motions ($\mu _{\alpha }\cos \delta$, $\mu _{\delta }$) and and parallaxes in addition to sky positions ($\alpha$, $\delta$), with a limiting magnitude of $G = 21$ mag. The Gaia DR3 dataset supplies astrophysical parameters for many celestial bodies, derived from measurements of parallaxes, broad-band photometry, and mean radial velocity spectra. The parallax errors in Gaia DR3 range from 0.02 to 0.07 milliarcseconds (mas) for sources with $G \le 17$ mag, increasing to 0.5 mas at $G = 20$ mag and up to 1.3 mas at $G = 21$ mag. Similarly, proper motion errors range from 0.02 to 0.07 mas yr$^{-1}$ for $G \le 17$ mag, reaching 0.5 mas yr$^{-1}$ at $G = 20$ mag, and up to 1.4 mas yr$^{-1}$ at $G = 21$ mag, see Fig. 2.

**Fig. 3:**
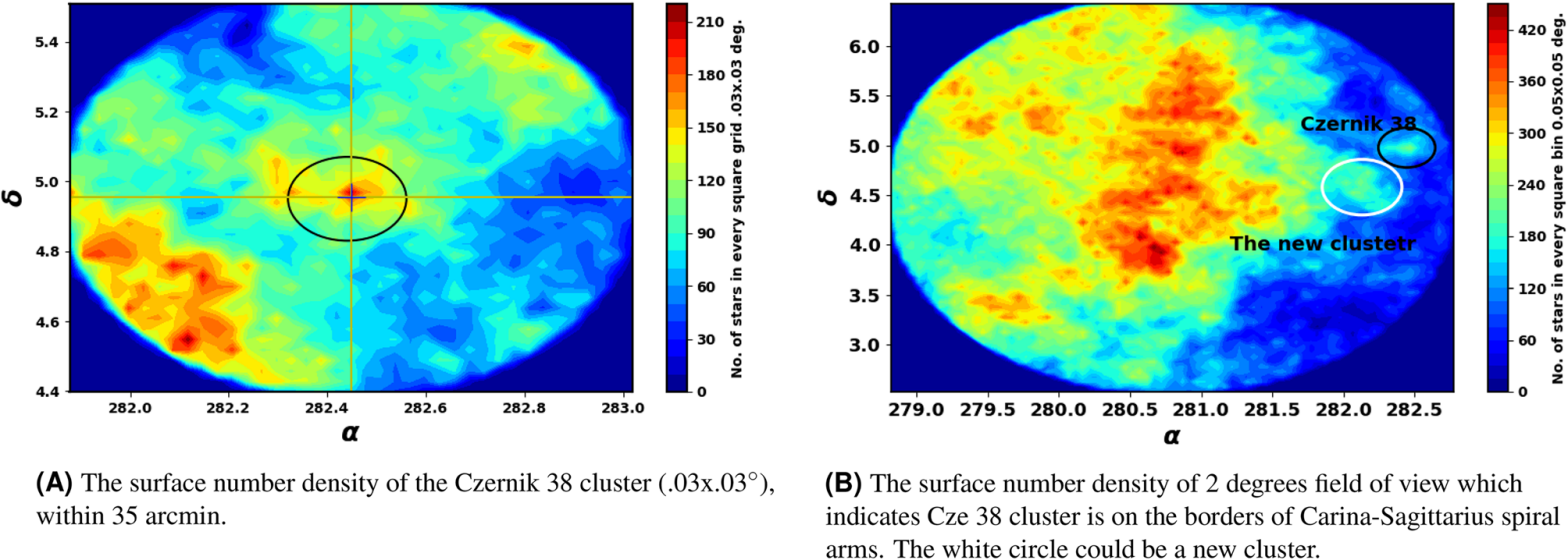
The surface number density of Czernik 38 using the data of Gaia DR3.

**Fig. 4:**
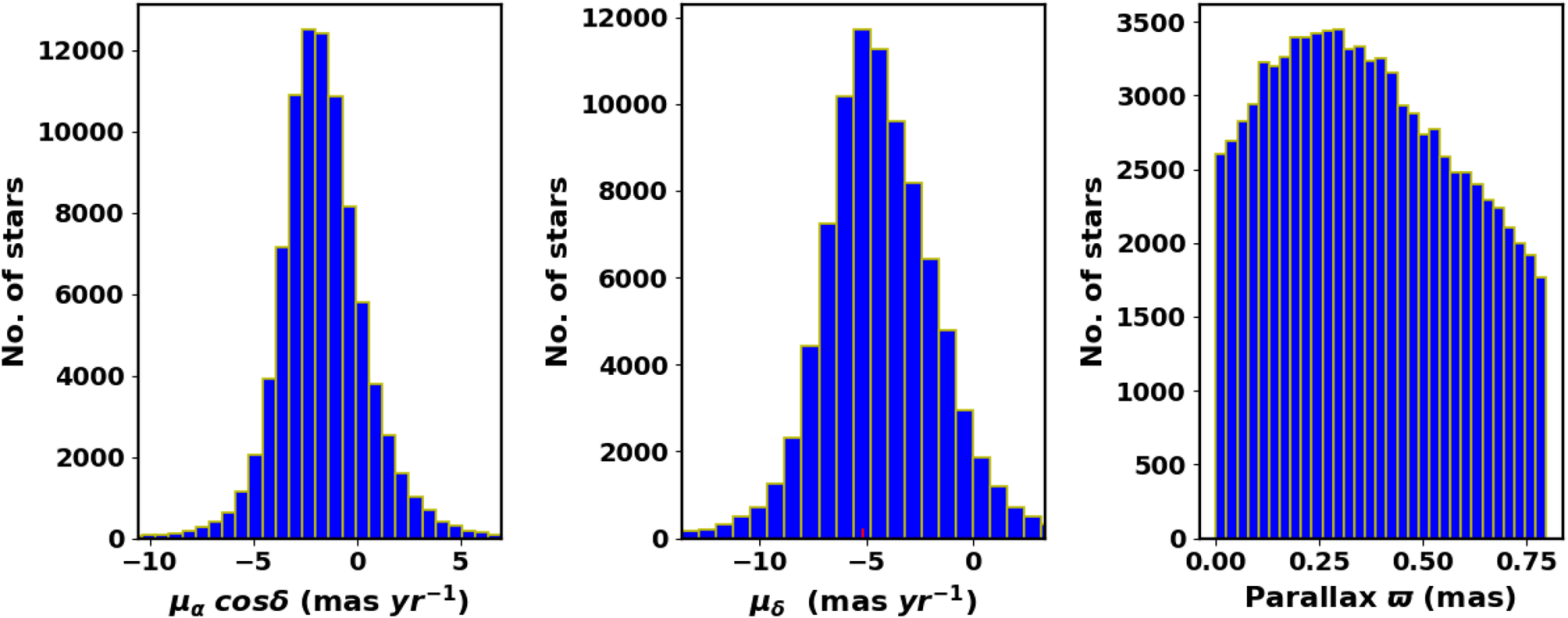
The proper motion in right ascension, declination, and parallax in the field of Czernik 38.

The catalog contains *G* magnitudes for approximately 1.806 billion sources, $G_{BP}$ magnitudes for around 1.542 billion sources, and $G_{RP}$ magnitudes for approximately 1.555 billion sources. Fig. 3 shows the surface number density of Czernik 38 derived from Gaia DR3, while Fig. 4 shows histograms of the proper motions ($\mu _{\alpha }\cos \delta$, $\mu _{\delta }$) and parallax ($\varpi$), in the field of Czernik 38.

### The data limits

**Fig. 5:**
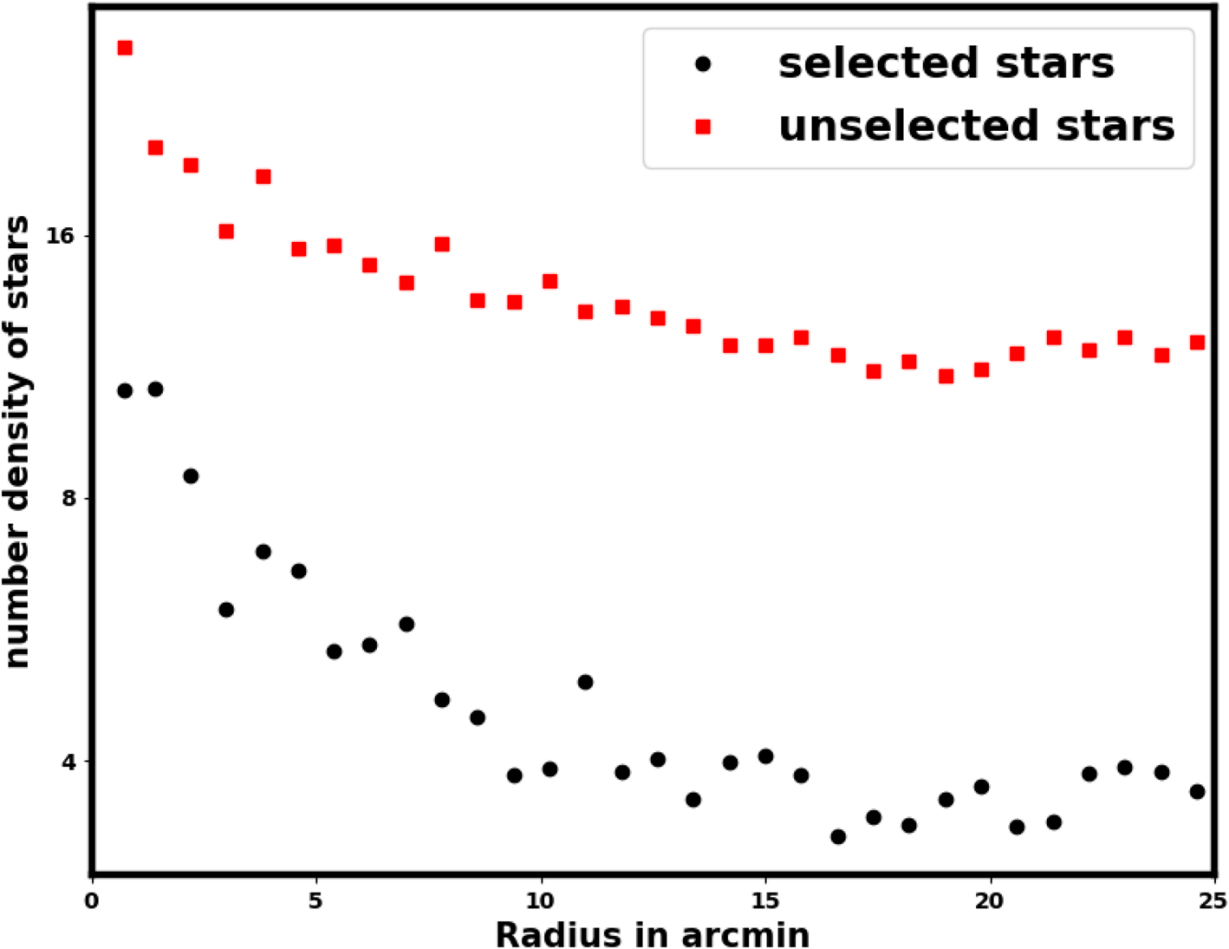
The black dots indicate the number stars density of selected stars, in case of the parallax condition $0.1 \le \varpi \le 0.35$, the red squares signify the stars that have not been selected.

While processing Gaia data it is important to note that inaccurate data clipping can lead to significant issues and erroneous outcomes when assessing cluster parameters such as core radius, the number of member stars, cluster mass, and overall cluster size, among others. Furthermore, the King model is capable of distinguishing the background level from member stars, which allows for minimal clipping. We restrict the Gaia data to a parallax range of 0.03 to 0.9 mas. The inappropriate clipping of data not only diminishes the field stars but also decreases the number of cluster member stars. For instance, in Fig.5, we illustrate the radial density profile (RDP) of unselected stars under the parallax condition $0.1 \le \varpi \le 0.35$.

This figure shows that the clipping data is mostly overdensity of member stars. Acquiring the RDP of unselected stars is essential for assessing whether any structural features or overdensity persist.

When computing averages for any variable or parameters influenced by random errors, it is essential to recognize that these averages are based on data that satisfies a relative error threshold of 10%. For instance, regarding the mean parallax, we set forth the following condition:

$$\begin{aligned} \frac{\mid \Delta \varpi \mid }{\varpi } \le 10\%, \; where \;\; \;\; \Delta {\varpi } = \varpi - \mu _{\varpi } \end{aligned}$$

where $\Delta \varpi$ is the parallax error in Gaia archive.

In contrast, if any parameter or variable shows features of a Gaussian distribution or is approximately similar to it:

1 $$\begin{aligned} N \approx N_o \; \exp \left( \frac{-\Delta \varpi ^2}{2\;\sigma ^2} \right) \end{aligned}$$

Consequently, it is frequently observed that values exhibit considerable inaccuracies. Therefore, we cannot eliminate the data; instead, we can apply the Gaussian function fit to calculate the mean value.

In our research, the distributions of parallaxes and proper motions exhibit a close resemblance to a Gaussian distribution. As a result, we implemented a minor truncation of the data, selecting parallax values that lie within the range of 0.03 to 0.9 mas. Furthermore, we computed the average values of parallaxes and proper motions by applying a Gaussian distribution fit to these datasets, see Fig. 16 in Section "The cluster dynamics and kinematics".

### 2MASS Data

The Two Micron All-Sky Survey (2MASS^28^;) utilized two automated 1.3m telescopes, located at Mt. Hopkins, Arizona (USA) and the Cerro Tololo Inter-American Observatory (CTIO) in Chile.Each telescope was equipped with a three-channel camera, which included a 256 $\times$ 256 array of HgCdTe detectors in every channel. The 2MASS catalog provides photometric measurements in the J (1.25 $\upmu$m), H (1.65 $\upmu$m), and K$_s$ (2.17 $\upmu$m) bands, covering millions of galaxies and nearly half a billion stars. The catalog’s sensitivity reaches magnitudes of 15.8 in J, 15.1 in H, and 14.3 in K$_s$ at a signal-to-noise ratio (S/N) of 10.

## Radial density profile and cluster structure

To analyze the cluster structure and construct the radial density profile (RDP), the first step is to accurately determine the cluster’s center. The primary objective is to locate the region with the highest stellar density. To achieve this, we generated a two-dimensional histogram of star counts in right ascension ($\alpha$) and declination ($\delta$) using data from the Gaia DR3 database. By utilizing the histogram2d function from the numpy package, we identified the cell containing the maximum number of stars. This method was conducted again in Section “Membership determination”, with a focus exclusively on member stars, revealing no significant differences. To determine the size of the cluster, we created the RDP of Czernik 38 by dividing the observed area into concentric rings. The number of stars in each ring, $N_i$, was counted, and the star density was calculated as $n_i = N_i / A_i$, where $A_i$ represents the area of the *i*-th ring ($\pi (R_{i+1}^2 - R_i^2)$). In this context, $R_{i+1}$ and $R_i$ represent the outer and inner radii of each ring, respectively, with the ring radius being defined as:

2 $$\begin{aligned} r_i = \frac{R_{i} + R_{i+1}}{2}. \end{aligned}$$

The stellar density function $n_{t}(r)$, which denotes the overall stellar density (comprising both field stars and members of clusters), is:

3 $$\begin{aligned} n_{t}(r) = n_{bg} + n_{c}(r), \end{aligned}$$

where the background star density is $n_{bg}$ and the cluster member stars density is $n_{c}(r)$.

One of the famous profile for cluster stars density that was created by King (1966) is as follows:

4 $$\begin{aligned} n_{c}(r) = \dfrac{n_{o}}{1+(r/r_{c})^{2}}, \end{aligned}$$

where $r_c$, $n_{bg}$, and $n_o$ denote the core radius, background density, and central density, respectively. The core radius, $r_c$, signifies the distance from the center of the cluster at which the stellar density, $n_{c}(r)$, reaches half of the central density $n_{o}$. An additional parameter, the limiting radius $r_{lim}$, was introduced by^29^. This radius is determined by comparing $n_c(r)$ in equation 4 to the background density threshold, $3 \sigma _{bg}$, defined as:

5 $$\begin{aligned} n_c(r_{lim}) = \dfrac{n_{o}}{1+(r_{lim}/r_{c})^{2}} =3 \sigma _{bg}, \end{aligned}$$

where $\sigma _{bg}$ is the uncertainty in $n_{bg}$. The limiting radius is then calculated as:

6 $$\begin{aligned} r_{lim} = r_c \sqrt{\frac{n_o}{3 \sigma _{bg}} - 1}. \end{aligned}$$

In Equation 4, the term for cluster radius is absent, which may occasionally result in imprecision. An alternative density function formula found in the literature incorporates this cluster radius, enhancing its accuracy, and it is:

7 $$\begin{aligned} n_t(r) = {\left\{ \begin{array}{ll} n_{bg} + k \left[ \dfrac{1}{\sqrt{1+(r/r_c)^2}} - \dfrac{1}{\sqrt{1+(r_{cl}/r_c)^2}} \right] ^{\beta }, & r \le r_{cl}, \\ n_{bg}, & r> r_{cl}, \end{array}\right. } \end{aligned}$$

and *k* is:

8 $$\begin{aligned} k = n_o \left[ 1 - \dfrac{1}{\sqrt{1+(r_{cl}/r_c)^2}} \right] ^{-\beta } \end{aligned}$$

and

$$\begin{aligned} n_c(r_c) = k \left[ \dfrac{1}{\sqrt{2}} - \dfrac{1}{\sqrt{1+(r_{cl}/r_c)^2}} \right] ^{\beta } \end{aligned}$$

This is an earlier version of the equation 4, which was proposed by^30^ ($\beta =2$). However, in this context, we will introduce the $\beta$ index in place of 2. We fit the RDP of the cluster with the equation 7, allowing $\beta$ to take on only the values 1 or 2.

**Fig. 6:**
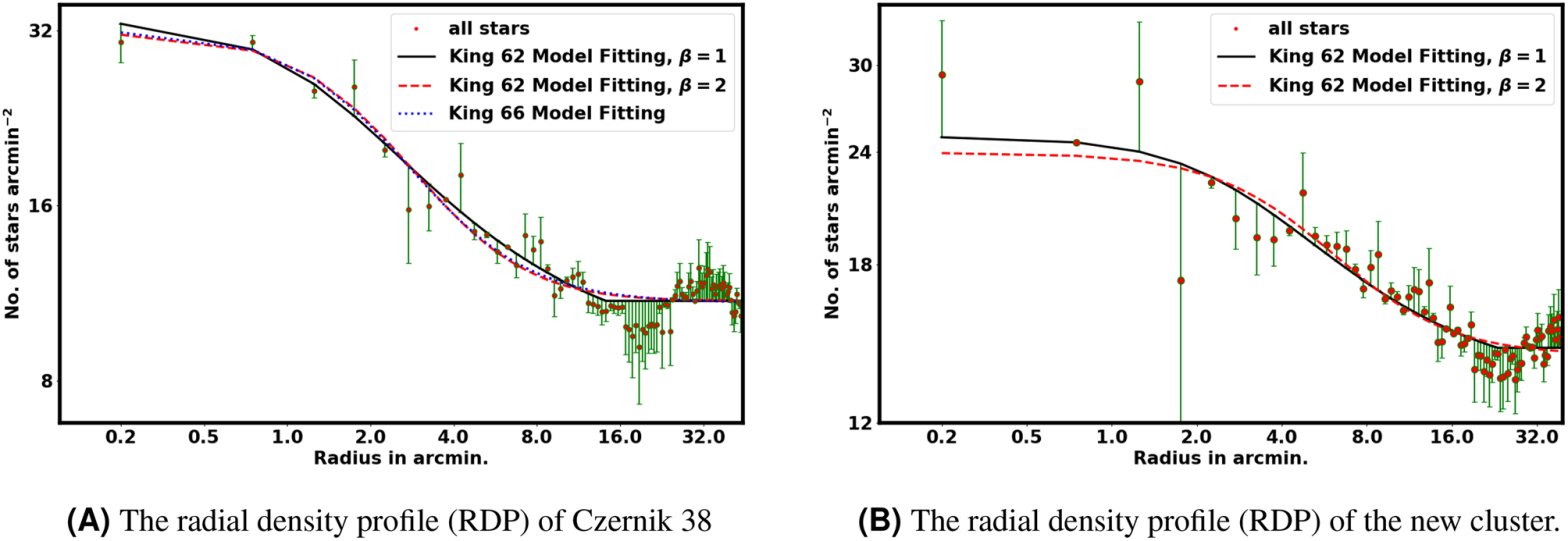
The radial density profile (RDP) of the two clusters. The solid black line and the dashed red line illustrate the King model fits with $B=1$ and $B=2$, respectively, while the dashed blue line corresponds to the King model^32^.

In this research, the optimal fit occurs at $\beta$ equal to 1; however, this is not universally applicable, as the fitting value is affected by the steepness of the density profile of the cluster. In some cases, $\beta$ can equal 2, leading to a better fit. The term $r_{cl}$ is a misnamed tidal radius in literature. But it is the cluster radius at which the cluster star density drops to zero. This model offers improved accuracy over Equation 4 (see Fig. 6), In addition, the Equation 7 can be very helpful for knowing the total number of stars in a cluster. The stellar density $n_{c}(r_i)$ in the *i*-th ring can be used to estimate the number of member stars in that ring:

9 $$\begin{aligned} N_{cl, i} = n_{c}(r_i) \cdot A_i, \end{aligned}$$

By summing the cluster density profile up to $r_{cl}$, we can estimate the total number of cluster members inside this radius:

10 $$\begin{aligned} N_{cl} = \sum _{r=0}^{r_{cl}} \left[ N_i - n_{bg} \cdot A_i \right] . \end{aligned}$$

This $N_{cl}$ term represents the total number of cluster members that can significantly limit the probability cutoff value, as it will be discussed in section "The probability cut-off value".

The structural parameters of Czernik 38 were determined by fitting the King model to the RDP. The background density, $n_{bg}$, was found to be 11.6$\pm$ 0.03 stars arcmin$^{-2}$ (indicated by the blue dashed line in Fig. 6). The central density, core radius, and cluster radius were determined to be 22.33$\pm$ 2.24 stars arcmin$^{-2}$, 1.19 $\pm$ 0.02 arcmin, and 14.36$\pm$ 7.63 arcmin, respectively (see Table 1). Furthermore, the total number of member stars $N_{cl}$ were estimated as 938 $\pm$ 61 stars. The uncertainties in the fitted parameters were estimated using the covariance matrix obtained from the curve_fit function in the scipy package (https://scipy.org/).

To quantify the compactness of Czernik 38, we calculated the star density contrast:

11 $$\begin{aligned} \delta _c = 1 + \frac{n_o}{n_{bg}}. \end{aligned}$$

For Czernik 38, $\delta _c$ was found to be 3.03 $\pm$ 0.02, significantly lower than the typical values for compact clusters ($7 \le \delta _c \le 23$) reported by^31^, based on the fact that Czernik 38 is a sparse cluster.

**Table 1:** King model fit parameters.

Ref.	$n_o$	$n_{bg}$	$r_c$	$r_{cl}/r_{lim}$
	No.$arcmin^{-2}$	No.$arcmin^{-2}$	arcmin	arcmin
This work, Eq. 7	22.33$\pm$2.24	11.6$\pm$0.03	1.19$\pm$0.02	14.36$\pm$7.63
This work, Eq. 4	22.91	14.56	2.56	4.2 ± 0.87
^7^	11.6 ± 0.6	15.0 ± 0.1	1.3 ± 0.09	4.0

## Membership determination

The assessment of fundamental parameters for star clusters is commonly complicated by the presence of field star contamination. Historically, the determination of cluster membership relied on photometric and kinematic data. In light of the astrometric data provided by the Gaia survey, the reliability of kinematic approaches for ascertaining membership has seen substantial improvement. Proper motion and parallax data are particularly effective in distinguishing field stars from cluster members, as stars in a cluster tend to share similar kinematic properties and distances^33^. In this study, we utilized Gaia DR3 proper motion and parallax data to identify cluster members.

### The membership method: HDBSCAN algorithm

We used the Unsupervised Photometric Membership Assignment in Stellar Clusters (UPMASK) algorithm, developed by^34^.This approach is a non-parametric and unsupervised, eliminating the necessity for prior selection of field stars. A refined version, available as the pyUPMASK Python package (https://github.com/msolpera/pyUPMASK)^35^, builds upon the initial algorithm by integrating various clustering techniques from the scikit-learn library (https://scikit-learn.org/stable/)^36^. This library includes more than a dozen different clustering methods for unlabeled data, which are all available to use in pyUPMASK, such as Hierarchical Density-Based Spatial Clustering of Applications with Noise (HDBSCAN), OPTICS, KMS, Gaussian Mixture Models (GMM) and Mini Batch K-means (MBK), their references are in^35^. This allows for more flexible analysis of unlabeled data.

In this study, we employed the HDBSCAN algorithm as described by^37^, which has been implemented in Python by^38^. The HDBSCAN algorithm is considered one of the fastest clustering algorithms currently available, representing an advancement over both DBSCAN and OPTICS. Notably, DBSCAN operates under the premise that the criteria for clustering, specifically the density requirement, is uniform across the entire dataset. Consequently, DBSCAN may encounter difficulties in effectively identifying clusters that exhibit varying densities. HDBSCAN overcomes this limitation by loosening the uniformity assumption and exploring various density levels via the creation of an alternative representation of the clustering problem. Furthermore, it primarily utilizes a k-means clustering algorithm, which is a technique for classifying data based on its closeness to specified center points. Moreover, It is effective at identifying and removing noise in a data set. Then, HDBSCAN is one of the most widely used and cited clustering algorithms.

**Fig. 7:**
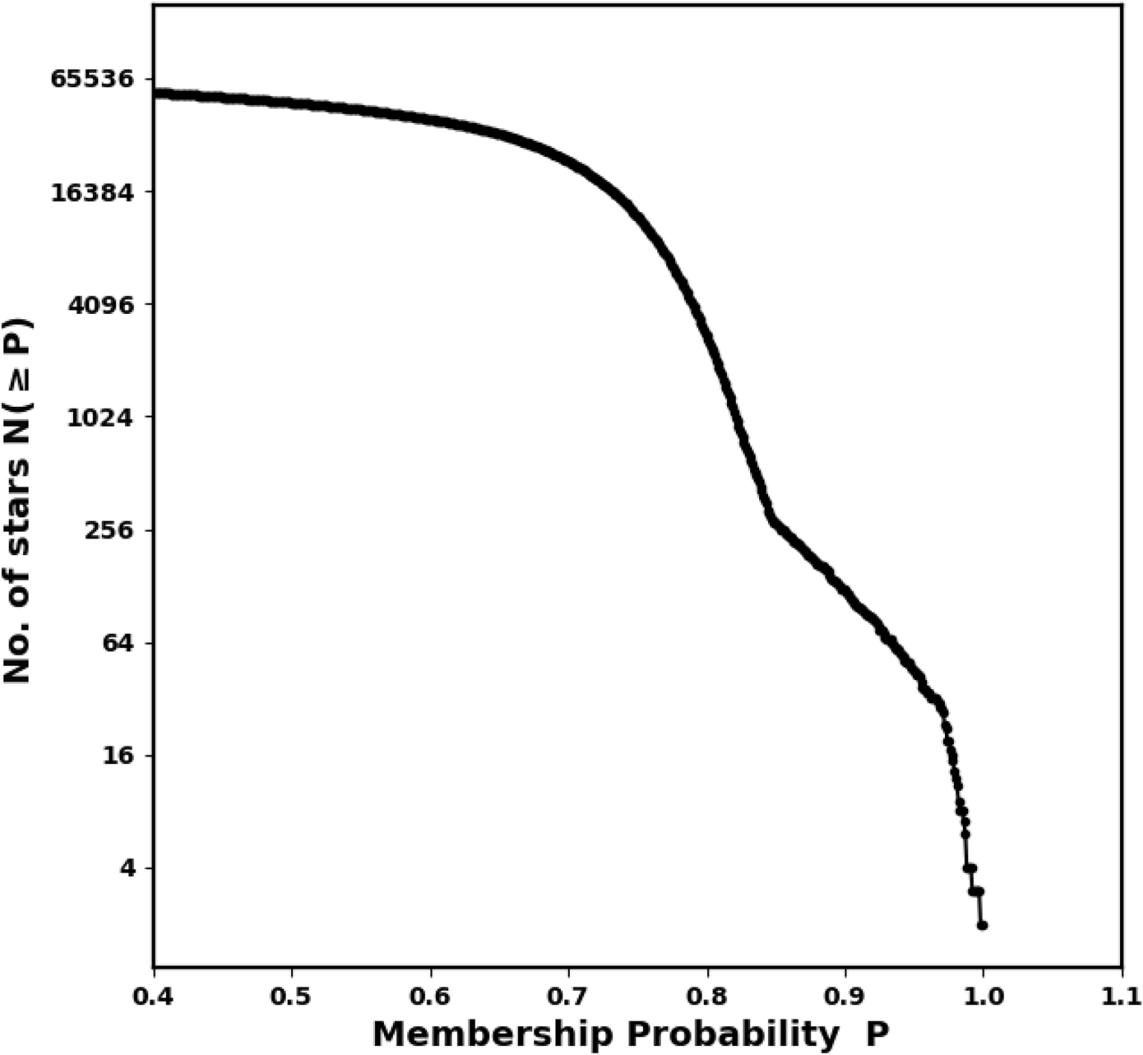
The number of stars as function of membership probability, the output of *pyUPMask* code.

In this research, we employed the pyUPMASK package alongside the HDBSCAN algorithm to determine the membership probabilities of stars located within the cluster. Gaia DR3 data ($\alpha$, $\delta$, $\mu _{\alpha } \cos \delta$, $\mu _{\delta }$ and $\varpi$) for approximately 130,238 stars within a $50^\prime$ radius were used as input. Fig. 7 shows the total number of stars, N($\ge$P), as a function of their probability of membership P.

### The probability cut-off value

A cut-off probability of 50% is frequently employed for determining membership; nevertheless, this threshold may not always be suitable. The appropriate cut-off is influenced by the method applied, as well as factors including the density of the surrounding field and the distance separating the star from the cluster center. Moreover, the optimal probability threshold may differ from one cluster to another. Thus, it is vital to thoroughly assess the determination of the probability cut-off, as an inaccurate threshold could cause the misclassification of members within the cluster. Recent investigations have adopted differing probability cut-off values. For instance^16^, implemented the same technique (HDBSCAN) as we did, with a probability cut-off established at 50%. On the other hand^39^, and^40^ applied UPMASK with a probability cut-off of P> 70% and the GMM model with P > 80%, respectively. Thus, the choice of the probability cut-off value remains a subject of ongoing debate. *Furthermore, in the majority of studies, the RDP of these member stars identified at this specific probability cutoff value does not align with the king profile, presenting a significant and crucial contradiction.*

**Fig. 8:**
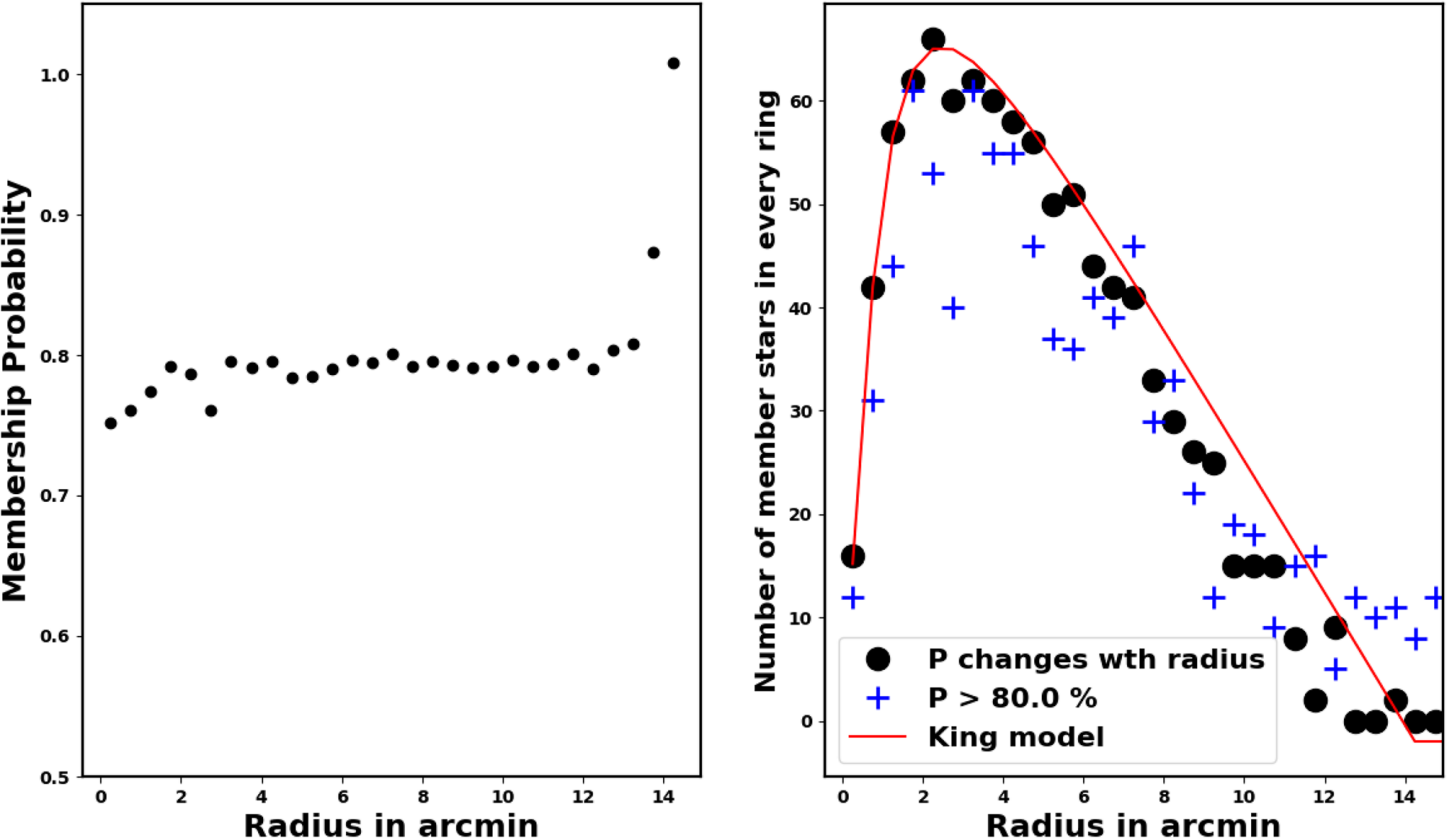
“Our methodology involves analyzing the Probability $P_{i}$ at every ring as a function of the radius $r_i$.

To tackle this problem in our study, we adopt the approach by using a radius-dependent cut-off value (see Fig. 8). The fitted King profile model also plays a significant role in this process. Each individual ring *i* has an associated probability $P_i$ that results in a stars number identical to the number of stars inferred from the King model fit $N_{cl,i}$, see equation 9. The approach is mathematically:

12 $$\begin{aligned} Np_{i}(P\ge P_i) \;\approx \; N_{cl,i} \end{aligned}$$

where $P_i$ is the probability in the *i*-th ring, giving the number of member stars as $Np_{i}$, which should match the number of stars from the King model, $N_{cl,i}$, as shown in Fig. 8. For example, if there are 100 member stars in shell *i*, as determined by the King fit, the probability cut-off values $P_i$ will also result in the same number, 100 stars. The left panel of Fig. 8 illustrates the relationship between $P_i$ and $r_i$. The indices of these member stars in ring *i* are provided in Python as:

$$\begin{aligned} Index_i \;=\; (\; P>= P_i \; ) \end{aligned}$$

where *P* denotes the probability values obtained from the pyUPMASK code.

The King density profile is a significant tool for confirming the reliability of the membership separation method and the complete number of cluster members. In opposition, an inaccurate membership separation technique or a faulty probability cut-off may lead to an overestimation or underestimation regarding the number of member stars.

**Fig. 9:**
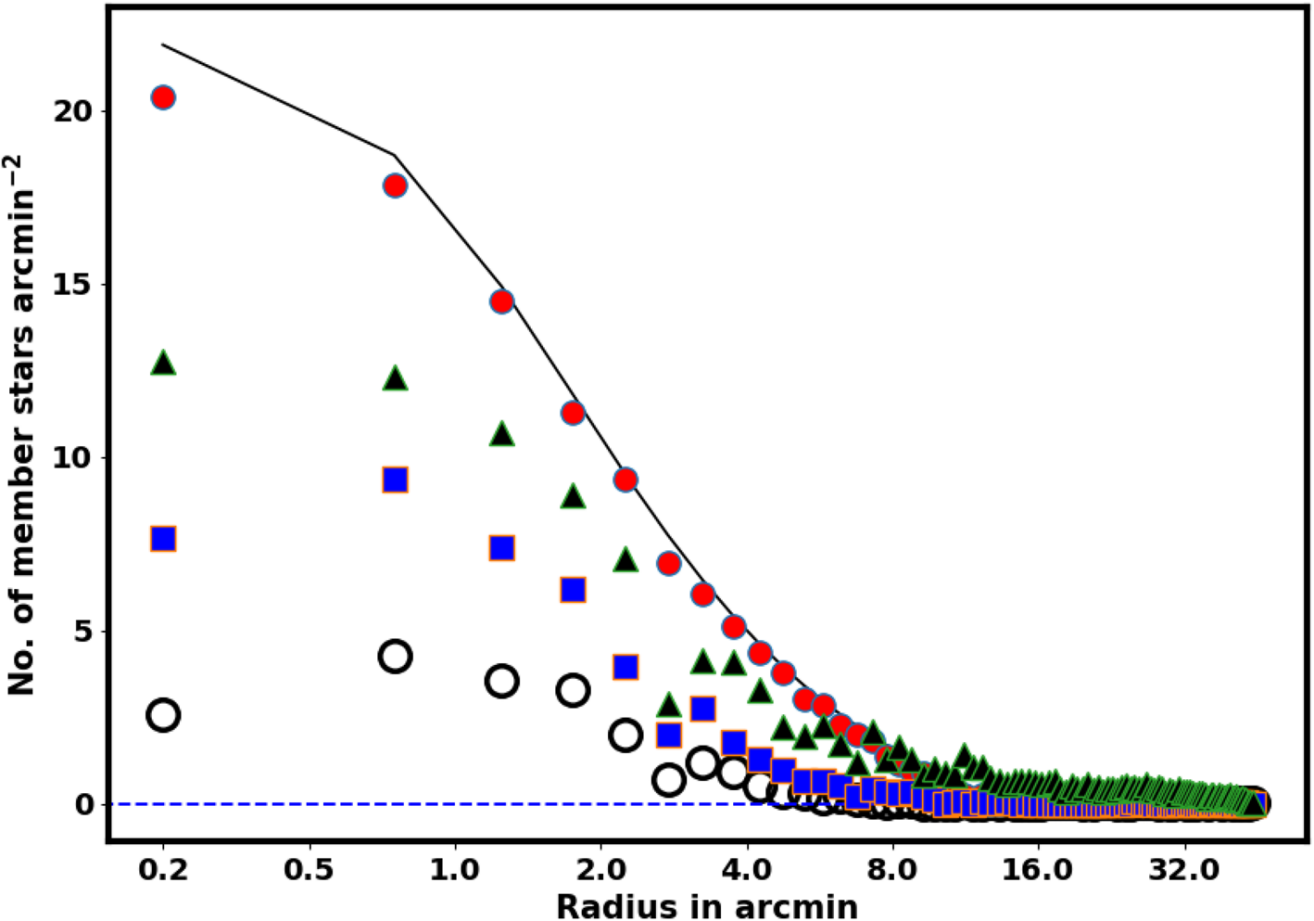
The density profile of member stars in the cluster is represented. The red dots are the Gaia DR3 probable member stars density, while the solid line illustrates the fitting of the King star density model for the cluster, derived from equation 7. The triangles are members from^41^ while the squares are members from^42^. The open circles are members from^43^. Neither of these groups follows the King model.

### What accounts for the difference in the number of member stars in comparison to others?

Our members’ findings have been compared with those in references^42,43^ and^41^, yielding a significant discrepancy; see Fig. 9. But what accounts for this? Thus, there are two explanations for this inconsistency.

The primary reason for the issue, which pertains to our membership technique, is to not associate the probability with the King profile, as explained in section 4.2. In other words, the members they found did not conform to their king model fitting, resulting in a fundamental contradiction that led to inaccuracy in assigning their members. This is despite the fact that the overdensities of stars are a crucial characteristic of OCs, and these overdensities often align with the King profile. The difficulty does not stem from the method itself, but from the decision regarding the probability cut-off value, which is supposedly what yields the King profile.

The second reason is the limitation of the data, as outlined in Section "The data limits". Inappropriate or excessive data trimming will reduce both field stars and members as well.

### The identification of a new open star cluster

Initially, during the preliminary data analysis, we perform a survey in two dimensions by establishing the surface number density (cell size is.05x.05$^{\circ }$), as illustrated in Fig. 3B. We have identified a clump of stars at the coordinates $\alpha =282.10^{\circ }$ and $\delta =4.56^{\circ }$, located 32 arcmin distant from Czerink 38. Additionally, its star number density closely aligns with the King profile, as illustrated in Fig. 6B, prompting us to consider the possibility that this may represent a new cluster.

**Fig. 10:**
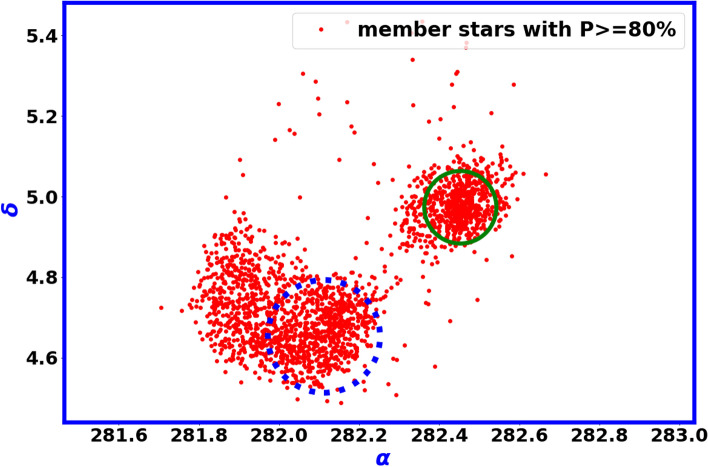
This diagram shows the distribution of member stars that have a probability greater than 80% within 50 arcmin from the center of Czerink 38. There are two groups of stars are found. The green solid circle represents the Czerink 38 area, whereas the dotted blue circle indicates a new cluster area.

In the second step, we create the probability map with $\alpha$ and $\delta$ within 50 arcmin, ensuring that the membership probability exceeds 80% to obtain members with a high probability, as illustrated in Fig. 10. This probability reveals the existence of two groups of stars, which indicates that they share nearly the same distance and proper motion. The green solid circle represents the Czerink 38 area, whereas the dotted blue circle indicates a new cluster area. It appears that they denote a binary cluster or a complicated colliding system within a dense concentration of stars and interstellar matter, and we aim to delve deeper into this captivating subject in future studies. Furthermore, regarding the photometric characteristics of this new cluster, it shares the same age, reddening, and distance as Czerni 38. For further details, please refer to the next Section "The photometry of Czernik 38 cluster".

## The photometry of Czernik 38 cluster

In the context of OCs, color-magnitude diagrams (CMDs) make use of empirical isochrones to evaluate against theoretical models of stellar evolution^44,45^. CMDs serve as effective tools for estimating key parameters such as distance, age, and metallicity of a cluster. Additionally, by comparing observed CMDs with theoretical isochrones, valuable insights into the masses of stars within the cluster can be obtained. The theoretical isochrones utilized in this research were obtained from the CMD 3.7 (http://stev.oapd.inaf.it/cgi-bin/cmd), using PARSEC version 1.25s^46^.

### Extinction

A precise interstellar dust extinction law is critically important for interpreting observations. The extinction coefficients for each passband depend on the source’s spectral energy distribution, interstellar matter, and the extinction itself. Both the color excess ratio (CER), $E(\lambda -\lambda _1)/E(\lambda _2-\lambda _1)$, and the relative extinction, $A_\lambda /A_{\lambda _1}$, are key indicators of the extinction law.

We follow the producer of^47^ and use the method presented in^48^, we compute the extinction coefficients in the Gaia, 2MASS and BV bands using the relation $A_{\lambda } = a A_V$. For example:

$$A_G/A_V = 0.789, \quad A_{BP}/A_V = 1.002, \quad A_{RP}/A_V = 0.589$$

For 2MASS bands:

$$A_J/A_V = 0.243, \quad A_{K_s}/A_V = 0.078, \quad A_H/A_V = 0.131$$

For the BV observations, we adopt the extinction law values from^49^ and^50^:

$$A_U/A_V = 1.558$$, $$A_B/A_V = 1.326$$, $$A_R/A_V = 0.81$$ and $$A_I/A_V = 0.56$$

By employing these values, the relationship between extinction and color excess can be delineated as follows:

13 $$\begin{aligned} {\begin{matrix} A_G & = 1.88 \times E(G_{BP} - G_{RP}) \\ A_V & = 3.1 \times E(B-V) \\ A_J & =1.473 \times E(J-K_s) \end{matrix}} \end{aligned}$$

The color excesses can be correlated with one another as follows:

14 $$\begin{aligned} {\begin{matrix} E(G_{BP} - G_{RP}) & = 1.29 \; E(B-V) \\ E(G_{BP} - G_{RP}) & = 2.5 \; E(J-K_s) \end{matrix}} \end{aligned}$$

Through isochrone fitting, we derive the color excess and, as a result, the extinction. The intrinsic distance modulus $(m-M)_0$ can be computed using the equation provided:

15 $$\begin{aligned} \left( m-M \right) _{\text {o}} = \left( m-M \right) _{obs} - A_{\lambda } \end{aligned}$$

where *m* is the apparent absorbed magnitude, *M* is the absolute magnitude, and $A_{\lambda }$ is the extinction in the $\lambda$ band. The expressions $(m-M)_o$ and $(m-M)_{obs}$ correspond to the intrinsic and observed distance modulus, respectively. Ultimately, we can derive the isochrone distance $d_{iso}$ as:

16 $$\begin{aligned} (m-M)_o \;=\; 5\; \log (d_{iso}) -5,\;\; \quad \quad \quad d_{iso} \;=\; 10^{\dfrac{(m-M)_o +5}{5}} \end{aligned}$$

### The color-magnitude diagram (CMD)

**Fig. 11:**
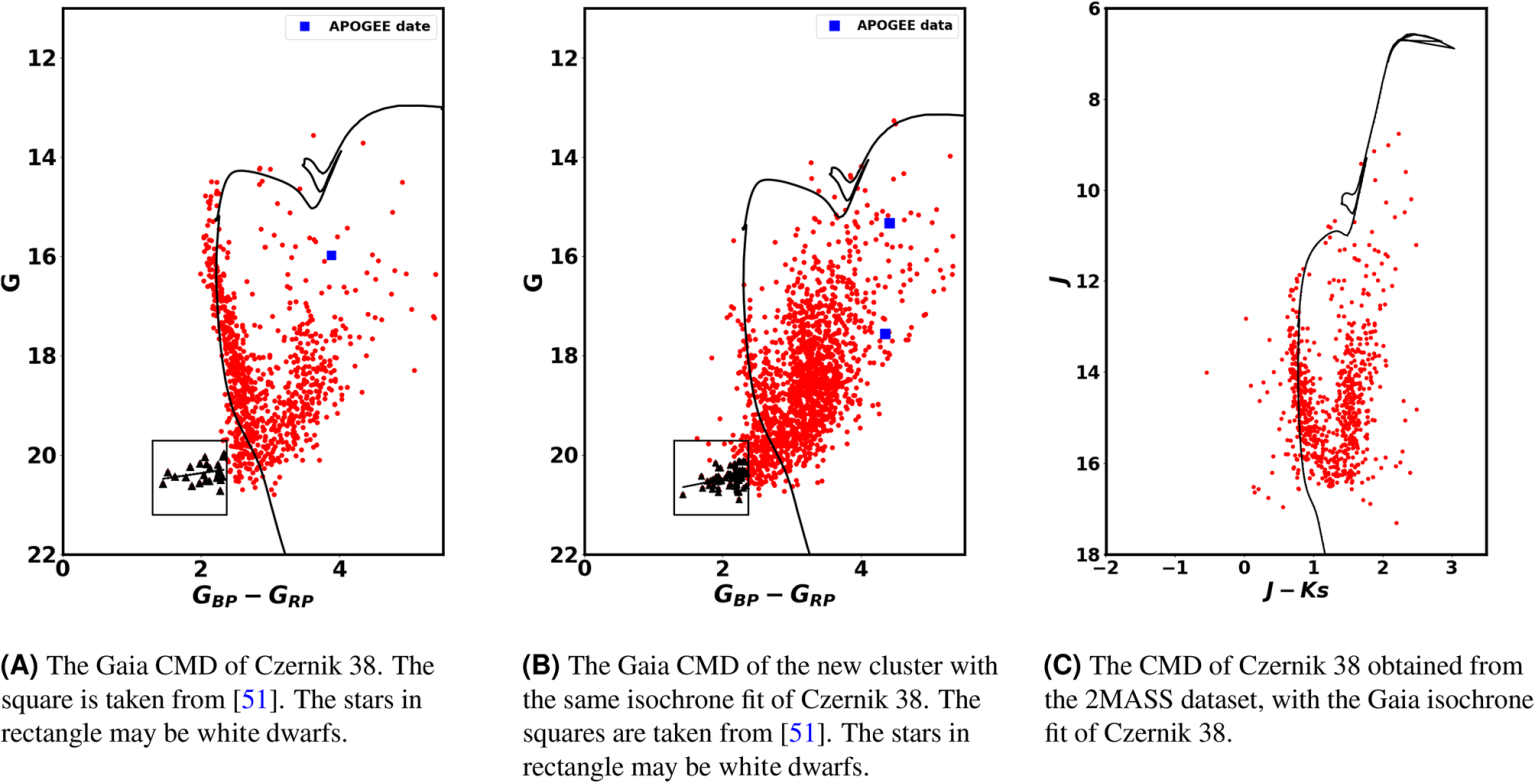
The Gaia and 2MASS CMDs of Czernik 38 and the Gaia CMD of the new cluster.

Using the photometric data from Gaia DR3 for stars in Czernik 38, the color-magnitude diagram (CMD) is presented in Fig. 11A. The CMD is fitted with theoretical isochrones from^44^. One of the numerous advantages of Gaia is its high precision in measuring parallaxes and distances. The geometric distance determined using Gaia data is 3580.4 $\pm$ 230.5 pc, please refer to Section "The proper motion, distance and cluster kinamtics" and see Fig. 16. This distance value limits the isochrone fitting, particularly when the cluster exhibits significant reddening. Only the young age isochrone yields this distance.

**Fig. 12:**
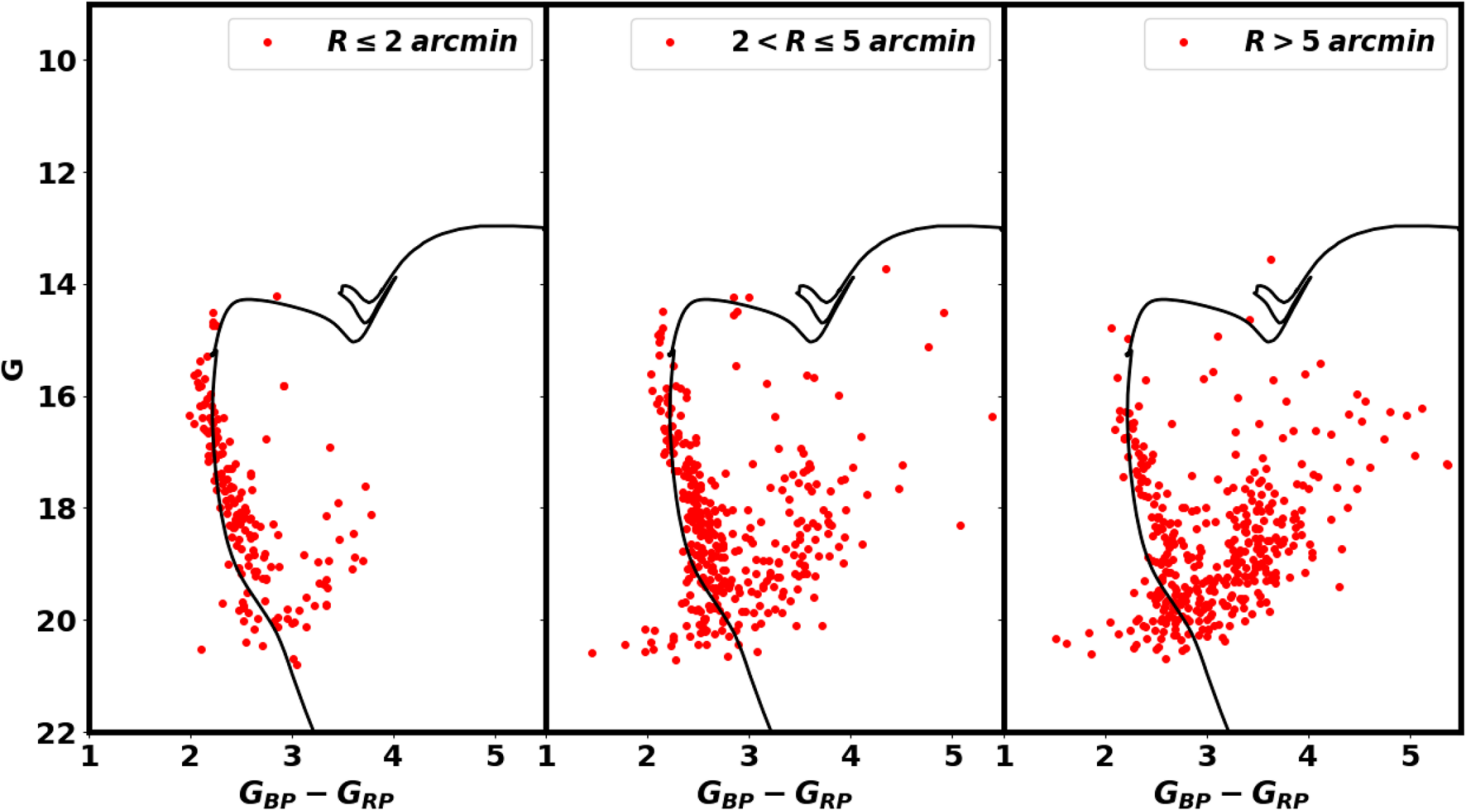
The color-magnitude diagram (CMD) for the Czernik 38 cluster was investigated at different radii to determine the homogeneity of reddening. The color excess $E(G_{BP}- G_{RP})$ is not the same in entire cluster.

We have determined that the intrinsic distance modulus and the color excess E(G$_{BP}$ - G$_{RP}$), are 12.69$\pm$ 0.08 mag and 2.40 $\pm$ 0.04 mag, respectively. The true distance modulus $\left( m - M \right) _{o}$ and the extinction in the G band ($A_G$) were calculated using the equation provided in Section “Extinction”. These results correspond to an isochrone-based distance $d_{iso}$ of 3448$\pm$ 362 pc. Furthermore, the fitted isochrone indicates a cluster age of approximately 115.0$\pm$ 20.3 Myr, with a metallicity of Z= 0.0152 ([M/H]= 0.0151 dex).

The CMD for the new cluster is depicted in Fig. 11B, with the same isochrone of Czernik 38. This suggests that the new cluster has comparable age, distance, and color excess to Czernik 38. Consequently, this could imply that both clusters are a binary system. We intend to investigate this fascinating topic further in our future research.

There is a notable inconsistency regarding the age and distance results in relation to the published values^7^. suggested that the cluster is at least 1 Gyr old and at a distance of 1.28 Kpc. On the other hand^8^, has concluded that the age and the distance are 600 Myr and 1.9 Kpc, respectively. Neither of them has a parallax value. The precise astrometry data from Gaia fundamentally altered our understanding of this cluster.

As depicted in Fig. 12, Gaia color-magnitude diagrams (CMDs) from various regions are employed to assess the consistency of interstellar material within the cluster and to investigate any potential structures as well. Despite its simplicity, this figure is of considerable importance. The investigation shows that the color excess $E(G_{BP}- G_{RP})$ is not uniform throughout the cluster. Overall, these figures are responsive to any phenomena associated with radius or position.

In order to construct the 2MASS CMD as illustrated in Fig. 11C, we first identified the member stars utilizing the Gaia astrometry and proper motion data. Subsequently, we correlated these stars with the 2MASS data, and thereafter, we applied the same isochrone depicted in Fig. 11A to fit the data. The distance modulus and the color excess $E(J-K_s)$ are determined to be 12.87 $\pm$ 0.93 mag and 0.89 $\pm$ 0.2 mag, respectively. The isochrone fits of the Gaia and 2MASS CMDs demonstrate remarkable consistency with minimal error. Furthermore, the ratio of $E(G_{BP}-G_{RP})$ to $E(J-K_s)$ satisfies equation 14.

Moreover, in relation to the photometric properties of this new cluster, we have fitted its data using the same isochrone as Czernik 38. The findings indicate that it possesses the same age, reddening, distance, and configuration as Czerni 38, suggesting the possibility that they may constitute a binary system.

### The right branch of CMD or red stars

On the right side of both Gaia and 2MASS CMDs, approximately one-third of cluster member stars form a branch that is nearly parallel to the main sequence, as depicted in Fig. 11. It is worth noting that these member stars are the result of the pyUPMASK, HDBSCAN techniques, indicating that they possess proper motion and distance characteristics similar to those of main sequence stars. Additionally, we have identified the same trend in our prior studies which did not include further discussion in these works, such as the King 18 cluster^52^ and the King 13 cluster^25^, *both of which are considered young clusters*. Thus, the existence of this type of star in these clusters might be linked to their young age rather than being coincidental or field stars. In this research, we will confront this issue and seek to confirm whether these stars are indeed part of the clusters or if they are field stars, using astrometric and physical methods. In any situation, we will obtain more details concerning these stars.

**Fig. 13:**
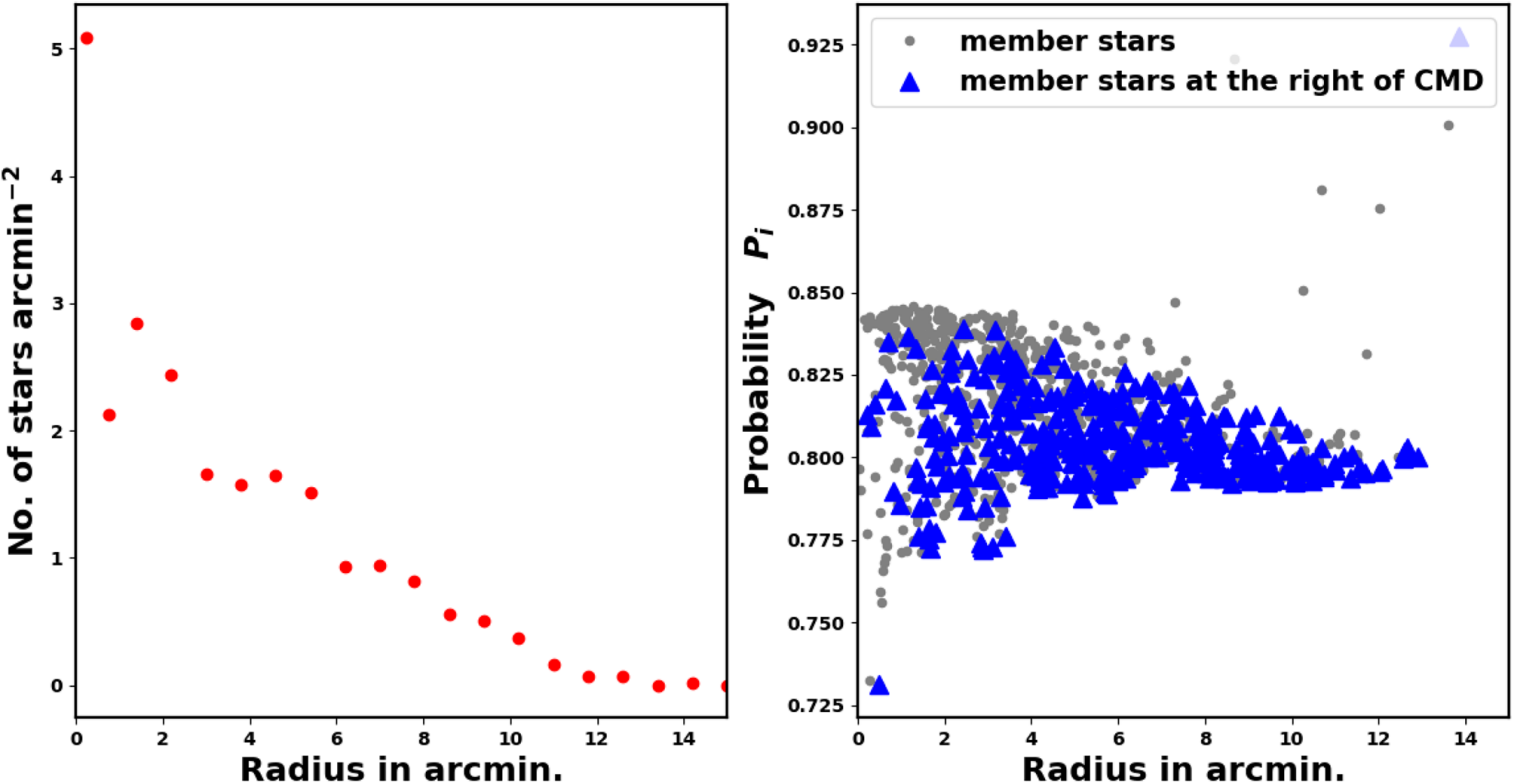
The left panel displays the RDP of stars located to the right of CMD. Meanwhile, the right panel illustrates the plot of probability in relation to the radius.

Initially, we can derive the RDP of these stars, as illustrated in the left panel of Fig. 13. This diagram indicates a distinct overdensity of these stars. Furthermore, we display the probability with respect to the radius, as depicted in the right panel of Fig. 13. This figure demonstrates that these stars possess high probability values. Consequently, the two panels illustrated in Fig. 13 suggest that these stars may be potential member stars, although this is not conclusive. Therefore, we will look for additional evidence.

**Fig. 14:**
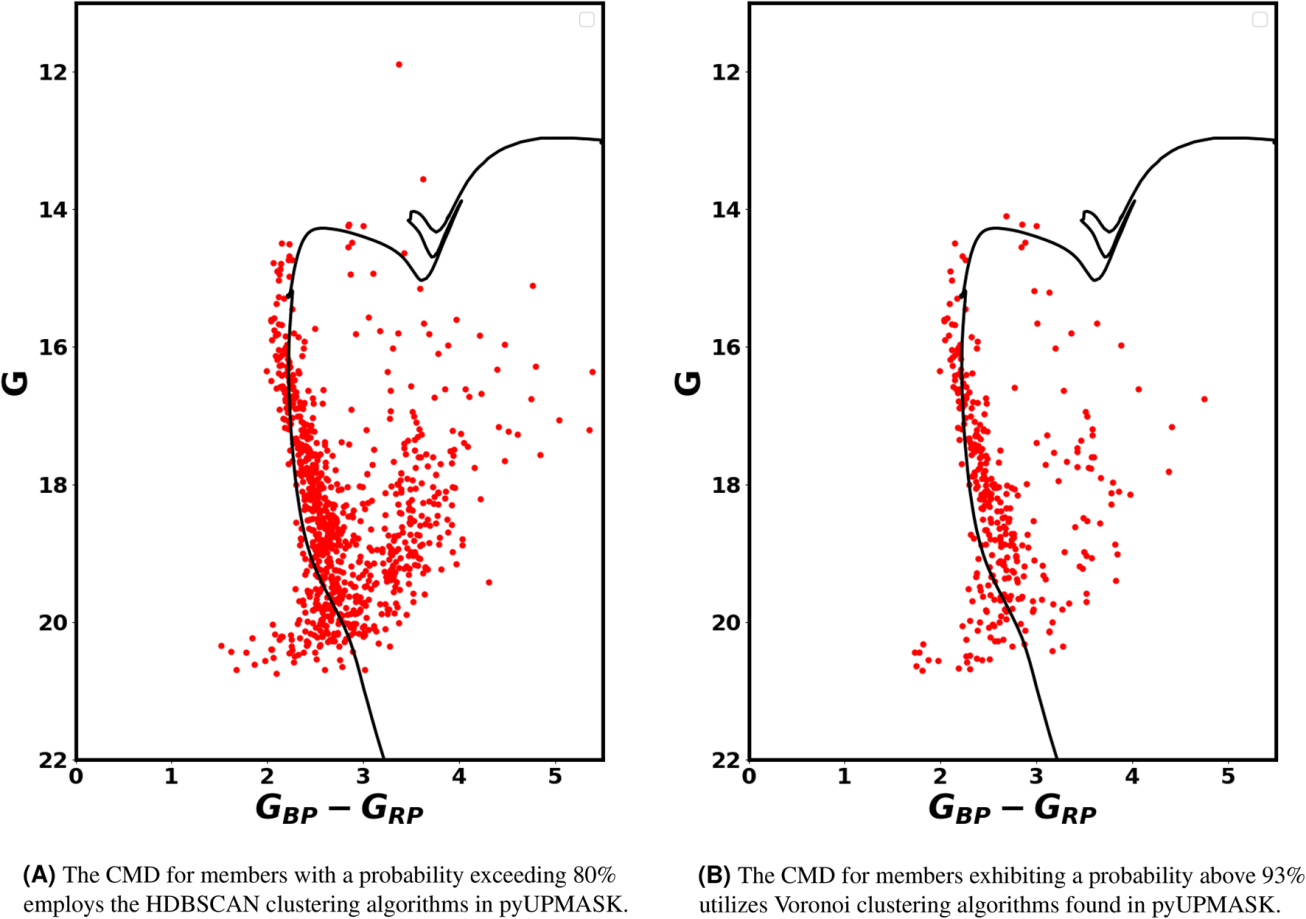
CMDs of the most probable members, using HDBSCAN and Voronoi technique. This figure shows that the right branch stars and faint blue stars among stars which are the most likely member stars rather than field stars.

We can generate the CMD diagram for the stars that are most likely to be members. Initially, we create the CMD for members whose probability exceeds 80%, utilizing the HDBSCAN clustering algorithm available in pyUPMASK, as illustrated in Fig. 14A. To further validate our results, we present the CMD using a different clustering approach, the Voronoi technique in pyUPMASK, for members whose probability surpasses 93%, as demonstrated in Fig. 14B. Consequently, the two clustering algorithms indicate that the majority of right branch stars, or red stars, are most likely members.

**Fig. 15:**
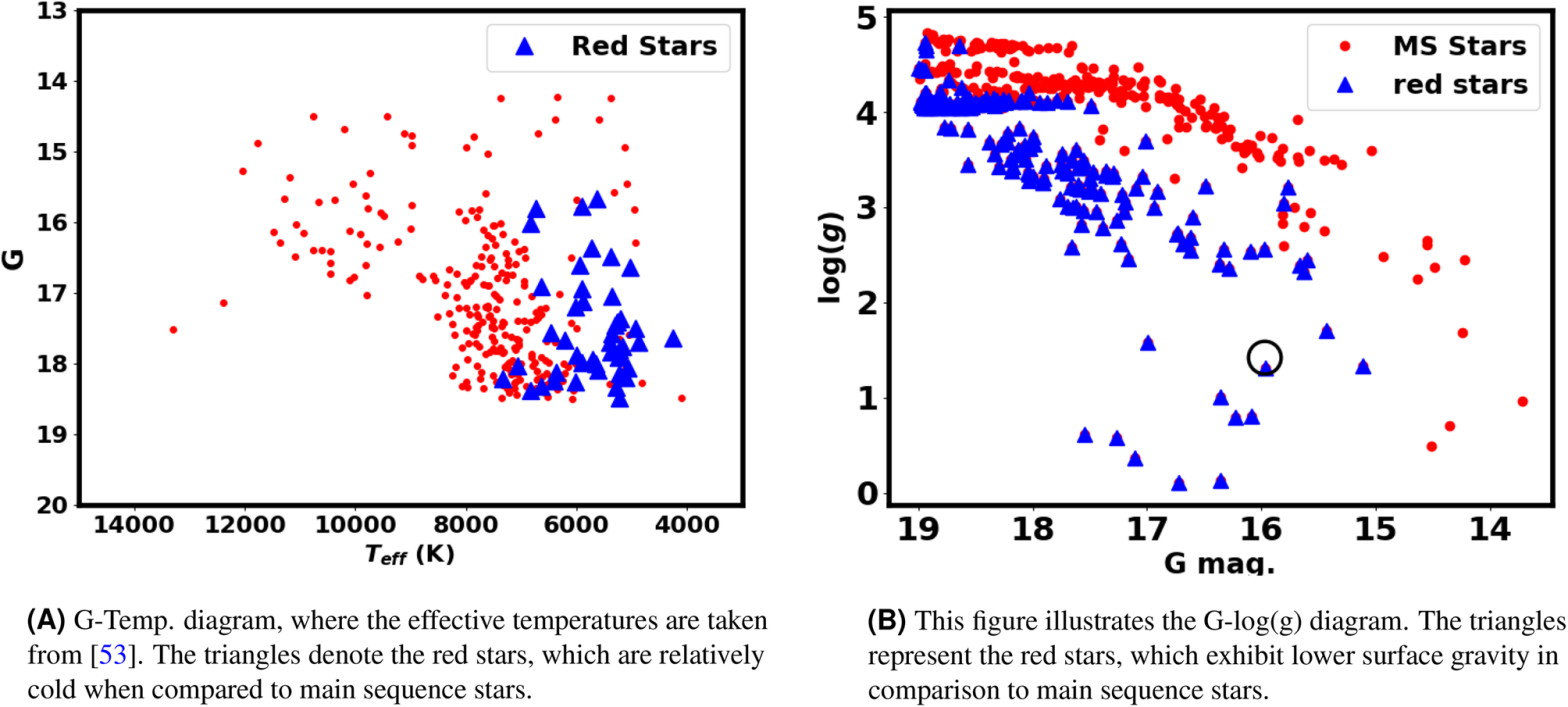
The triangles represent the red stars, which are distinguished by their reduced surface gravity and cooler environments compared to main sequence stars. This indicates that they are pre-main sequence stars rather than field stars.

With respect to the physical parameters such as effective temperatures and surface gravity, Fig. 15A presents the G magnitudes plotted against the effective temperatures, which are obtained from^53^. This impressive figure indicates that the stars on the right branch are cooler compared to the main sequence stars. Although these stars are relatively cool, but they have luminosities comparable to that of main sequence stars. This denotes that they have large surface areas. That result is consistent with the lower surface gravity detected by Gaia GSP-Phot for these right branch stars when compared to main sequence stars, as represented in Fig. 15B. Regardless of the accuracy, there is a clear general trend.

Moreover, we have matched our members with APOGEE-2 DR17 data^51^ One star has been matched, depicted as a square in Fig. 11A, exhibiting temperature, distance, and log surface gravity measurements of 4071.3K, 4607 pc, and 1.427, respectively, which are in agreement with prior results. Furthermore, the newly identified cluster contains two stars that are represented as squares in Fig. 11B.

As a result of previous, the hydrogen burning phase in these stars has yet to begin. These stars could be classified as type of pre-main sequence stars. This suggests a significant rate of star formation, which aligns with the notion of being a young cluster. It is unreasonable for a young star cluster situated in a dense gas region to lack any stars undergoing formation. In contrast, this characteristic of high star formation in this cluster is consistent with its young age, its high reddening value, and its unique location in the high density of stars and dense gasses.

In addition, we would like to emphasize that the right branch stars or the red stars are found in the new cluster as well, but with a higher ratio than that of the Cernik 38 cluster. Thus, it is crucial that we examine the new cluster more thoroughly and in greater detail.

In summary, this study has effectively shown that the right branch stars or red stars are part of the Czernik 38 cluster, while also emphasizing certain characteristics of these stars. However, it is crucial for these stars to undergo spectroscopic analysis to investigate this matter in greater detail. This procedure adds further depth and enhances the research concerning star formation and their underlying physics.

### The faint blue stars

At present, there are two primary methods for determining the age of stellar populations: the main-sequence (MS) evolution theory, which utilizes cluster isochrones, and the white dwarf (WD) cooling theory. A white dwarf found in a young open cluster is a stellar remnant that has developed from a massive progenitor star in a relatively young star cluster, which constitutes a rare but important find for astrophysicists. Then, the existence of white dwarfs within these relatively young clusters offers significant insights for research on the stellar evolution.

In current work, we have found that a number of stars appear to be fainter and bluer than the main sequence stars; see Table 2, which are indicated by stars in the rectangle in Fig. 11A. Additionally, they can be also found in the newly formed cluster as shown in Fig. 11B. Moreover, these stars are most probable member stars, as indicated in Fig. 14. These stars could potentially be young white dwarfs based on their locations in the Color-Magnitude Diagram (CMD). Interestingly, the correlation between *G* magnitude and the color $G_{BP} - G_{RP}$ is nearly the same in both clusters, as seen in Fig. 11, particularly since they share the same age and the same place. It is important to note that these stars are found beyond a radius of 2 arcmin, as depicted in Fig. 12. Moreover, the same pattern is found in the young open clusters Stock 12 and ASCC 113^54^.

**Table 2:** List of descovered white dwarfs.

	Gaia DR3 ID	$\alpha$	$\delta$	radius	G	$G_{BP}-G_{RP}$	Prob.
		Deg.	Deg.	Arcmin	Mag.	Mag.	Percentage (%)
1	4282131257964030720	282.488	4.897	4.269	20.178	2.074	80.20
2	4282131532836314368	282.467	4.905	3.113	20.351	2.250	81.61
3	4282142184355749760	282.322	4.878	8.248	20.184	2.247	81.57
4	4282142218715486080	282.337	4.890	7.104	20.494	2.284	80.17
5	4282143112068635264	282.388	4.924	3.470	20.455	2.230	81.43
6	4282143283872510720	282.463	4.921	2.219	20.576	1.449	78.82
7	4282143627464635904	282.431	4.947	0.575	20.514	2.114	76.58
8	4282144039781979008	282.318	4.935	7.362	20.037	2.054	80.31
9	4282144653966298112	282.402	4.976	2.771	20.719	2.274	78.22
10	4282144761336523008	282.393	5.002	4.206	20.438	1.777	82.55
11	4282147716274076800	282.385	5.004	4.615	20.561	1.976	80.48
12	4282225300559148800	282.552	4.942	6.716	20.426	1.622	80.75
13	4282225468068170496	282.534	4.939	5.677	20.343	1.519	80.25
14	4282237154668950144	282.485	4.942	2.730	20.282	2.259	77.61
15	4282237257748183424	282.487	4.959	2.887	20.513	2.059	80.67
16	4282238013662465280	282.507	5.002	5.051	19.970	2.331	81.08
17	4282238322900207616	282.422	4.989	2.569	20.165	1.980	76.15
18	4282238666497611648	282.433	5.013	3.830	20.398	2.037	80.48
19	4282239005803014144	282.491	5.036	5.990	20.018	2.312	80.30
20	4282239010094969856	282.487	5.040	6.073	20.617	1.868	80.34
21	4282239319332654336	282.487	5.069	7.649	20.250	2.129	80.55
22	4282240796801415296	282.493	5.080	8.429	20.235	1.845	81.79
23	4282242033752085376	282.377	5.061	7.659	20.433	2.305	79.58

**Table 3:** Some parameters of Czernik 38,, compared to others.

$\alpha$	$\delta$	$\mu _{\alpha }cos\delta$	$\mu _{\delta }$	${\varpi }$	N	age	$r_{50} / r_{cl}$	Ref.
deg.	deg.	mas yr$^{-1}$	mas yr$^{-1}$	mas	Stars	log (Gyr)	deg.	
282.45$\pm$0.05	4.97$\pm$0.05	−2.41$\pm$0.328	−5.263$\pm$1.063	0.21$\pm$0.083	938$\pm$61	115.0$\pm$20.3	14.36$\pm$7.63 arcmin ($r_{cl}$)	This work
282.444	4.963	−1.829 ±0.0.22	−4.984±0.23	0.377 ±0.137	151	8.614	0.038	^55^
282.451	4.965	−1.840	−5.005	0.383	194	-	0.041	^56^
2282.453	4.962	−1.777	−5.056	0.369	308	-	0.0485	^42^

## The cluster dynamics and kinematics

OCs are outstanding markers for tracing the evolution of the Galactic disc. The release of Gaia DR3 allows for the investigation of their Dynamics and Kinematics with an unprecedented level of precision and accuracy. The center of the cluster is located at 282.45 $\pm$ 0.05 and 4.97 $\pm$ 0.05, which corresponds to the Galactic coordinates l= 37.17 $\pm$ 0.05 $^\circ$ and b= 2.63 $\pm$ 0.05 $^\circ$.

### The proper motion, distance and cluster kinamtics

**Fig. 16:**
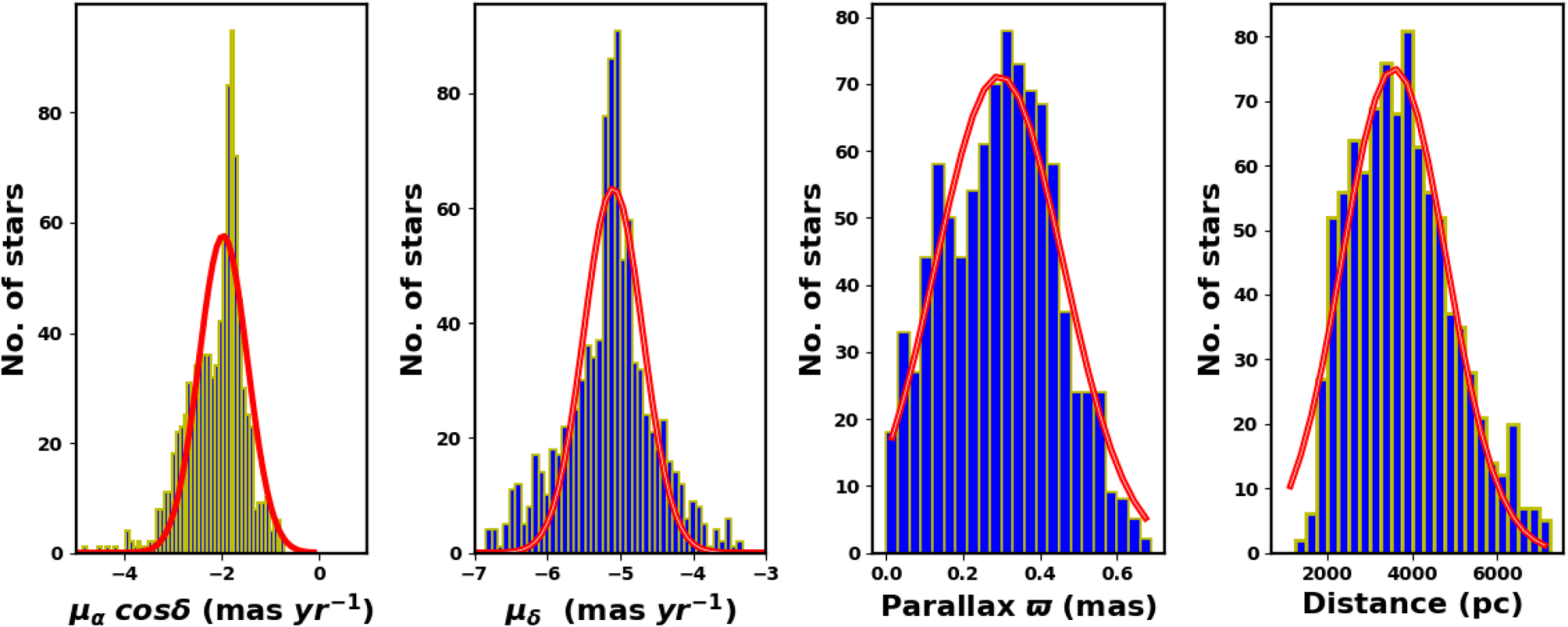
The members proper motions, parallaxes and distances histograms. The solid red lines are Gaussian fits.

The components of proper motion and parallaxes are modeled using Gaussian distributions, as illustrated in Fig. 16. The average values have been determined to be $\mu _{\alpha } \cos \delta$ = −2.41 $\pm$ 0.328 mas yr$^{-1}$, $\mu _{\delta }$ = −5.263 $\pm$ 1.063 mas yr$^{-1}$ and $\varpi =0.221$, respectively. To enhance the accuracy of parallax measurements, the parallaxes are adjusted according to the methodology outlined in^57^, implemented through Python code (gaiadr3_zeropoint). After applying a Gaussian distribution to the resulting histogram, Fig. 16 reveals that the mean parallax ($\varpi$) is 0.21 $\pm$ 0.083 mas. The detailed results are presented in Table 3. Table 3 also presents a comparison between our results and previously published values, showing good agreement overall.

Parallaxes ($\varpi$) play a crucial role in ascertaining distances; however, they do not directly convert to distances. This is due to the nonlinear relationship that exists between them, as well as the measurement noise that impacts distant stars. Even slight absolute errors in parallax may cause substantial uncertainties in distance calculations. In addition, while parallax can result in negative values, distances are not able to achieve this. A more effective method might involve using an explicit probabilistic approach to estimate distances^58^. provide distances catalog of 1.47 billion stars in Gaia EDR3, using probabilistic approach. Also, we fit the histogram of these members distances with Gaussian distribution. The mean distance to the cluster is found as 3580.4 $\pm$ 230.5 pc, see Fig. 16. This value is consistent with the results obtained from photometric data within the estimated errors.

It is well-known that the stars in the cluster move at almost the same speeds. The tangential velocities of OCs, calculated from absolute proper motions ($\mu = \sqrt{ (\mu _{\alpha }\cos \delta )^2 + (\mu _{\delta })^2}$) and parallaxes ($\varpi$) or distances, allow identification of the type of orbit the cluster follows. This contributes significantly to studies of cluster origins and destruction processes. The tangential velocity in km $s^{-1}$ is given by:

17 $$\begin{aligned} v_t = 4.74 \; \mu \; d \; \end{aligned}$$

where the constant 4.74 comes from the unit conversion:

$$\begin{aligned} \dfrac{(4.84 \times 10^{-6} \; \text {rad}) \; (3.086 \times 10^{13} \; \text {km})}{(3.154 \times 10^{7} \; \text {s})} \approx 4.74 \end{aligned}$$

**Fig. 17:**
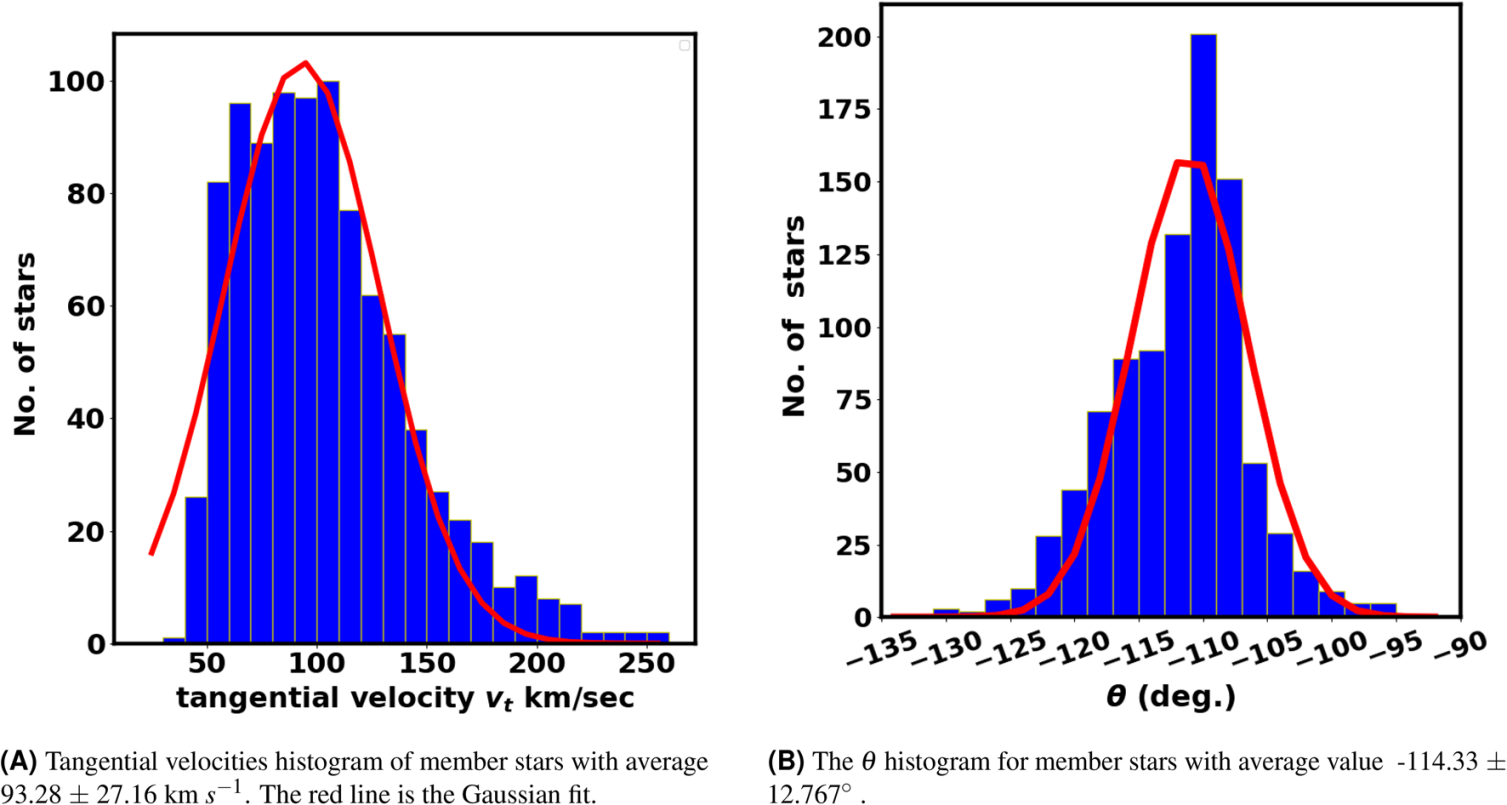
Tangential velocities and the $\theta$ histograms. The red line is the Gaussian fit.

Here, *d* and $\mu$ are the distance, proper motion in parsecs, arcseconds yr$^{-1}$, respectively. Fig. 17A shows a histogram of tangential velocity $v_t$ with an average value of 93.28 $\pm$ 27.16 km $s^{-1}$, following a nearly Gaussian distribution.

As cluster members generally move in nearly the same direction through space. The proper motions of the stars seem to converge at a singular point in the sky, which is called the convergent point. This apparent convergence is a perspective effect that arises from the shared trajectory of stars through space. As a result, grasping the tangential velocity alone does not suffice. A key supplementary parameter is the angle $\theta$, which indicates the direction of the cluster’s motion within the $\mu _{\alpha } \cos \delta$ and $\mu _{\delta }$ space. It is as described by this formula:-

18 $$\begin{aligned} \theta = \tan ^{-1}\left( \frac{\mu _{\delta }}{\mu _{\alpha } \cos \delta } \right) \end{aligned}$$

Fig. 17B presents a histogram of $\theta$ for member stars, with an average angle of −114.33 $\pm$ 12.767$^\circ$, providing a clearer view compared to Fig. 19. Additionally, the dispersion in the $\theta$ histogram could reflect the cluster’s age and its degree of gravitational binding. Also this dispersion cloud help test the different membership methods.

Furthermore, there are 37 member stars that exhibit radial velocities with an average value of 46.1 $\pm$ 8.54, in the Gaia DR3 archive. This value agrees with the value of^59^ (57.58 km $s^{-1}$.) within the error. So, the space velocity of the cluster ($v_{space} = \sqrt{v_r^2 + v_t^2}$) is approximately 109.8$\pm$ 13.56 km $s^{-1}$, making an angle of 25.5 $\pm$ 8.0$^{\circ }$ with tangential velocity direction. Then we can get the orbital parameters of the studied cluster, see next subsection “Cluster orbit”.

### Cluster orbit

OCs are remarkable markers for understanding the evolution of the Galactic disc. Utilizing Gaia DR3, researchers can analyze their kinematics with exceptional precision, particularly in terms of proper motion and parallax ($\mu _{\alpha } \cos \delta$, $\mu _{\delta }$, and $\varpi$). Furthermore, Gaia DR3 offers radial velocities (RV) for millions of relatively luminous, late-type stars^60^, gathered by the Radial Velocity Spectrometer (RVS) instrument^61^. The integration of parallax, proper motion, and RV yields significant phase-space data. For example^62^, demonstrated the great potential of Gaia data for studying the kinematics of the Galactic disc and OCs, revealing the richness of phase-space substructures. OCs trace the formation and evolution of our Galaxy. Their ages cover the entire lifespan of the Galactic disc, spanning the young to old thin-disc components. Their spatial distribution and motion help to better understand the gravitational potential and the perturbations acting on the structure and dynamics of the Galaxy. The orbital motions of OCs are essential not only for understanding their dynamical evolution in the Galaxy but also for investigating the dynamics of the Galaxy itself.

To compute a cluster’s orbit, we must first adopt a model for the Galactic potential. This potential must accurately reproduce the observed mass density of the Galaxy. For this purpose, we performed backward orbital integration of Czernik 38 using the **“MWPotential2014”** model, the default Galactic potential in the *galpy* package^63^. This model is made up of three parts: **(1)** the bulge component, described by a spherical power-law potential^63^, **(2)** the Galactic disk potential, defined by the Miyamoto-Nagai expression^64^, and **(3)** the dark matter halo potential, given by the Navarro-Frenk-White profile^65^. The Sun’s galactocentric radius, orbital velocity, and *z*-coordinate were taken as $R_{GC}=8$ kpc, $V_{rot}=220$ km $s^{-1}$, and $z=20.8$ pc^63^.

**Fig. 18:**
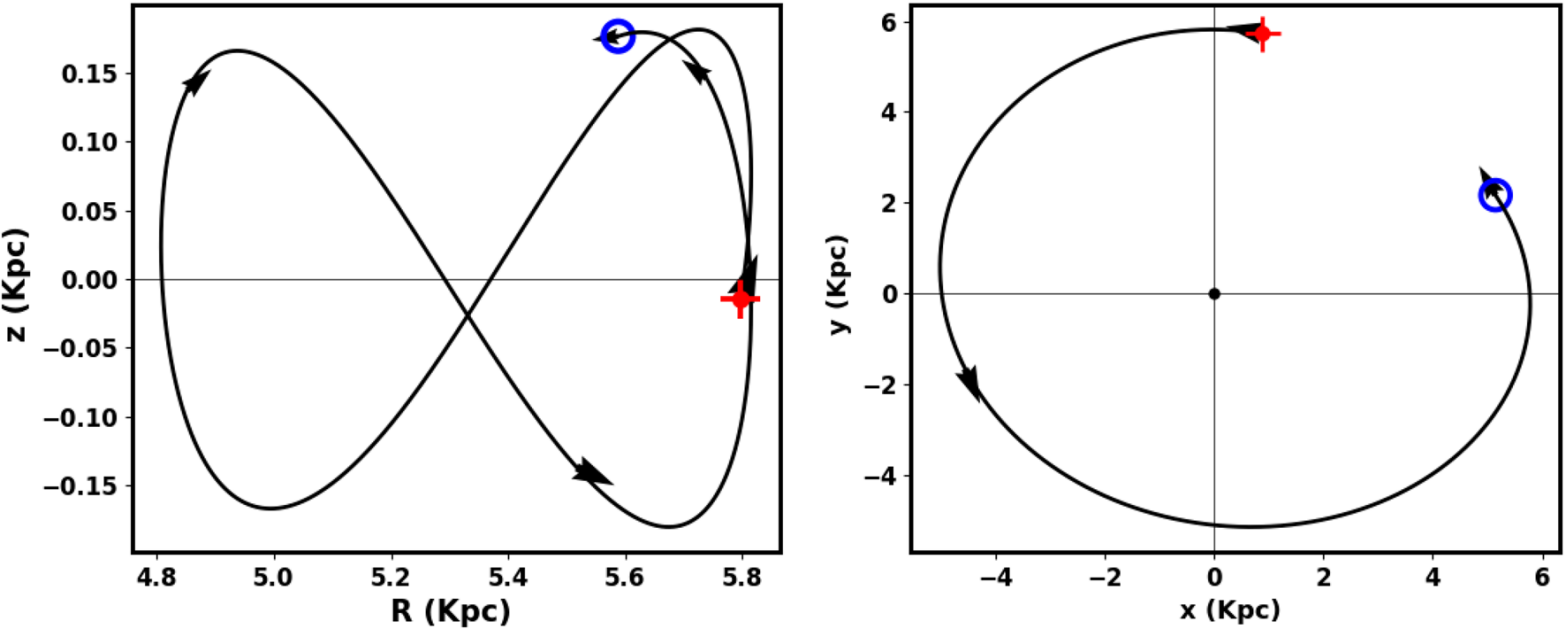
The cluster orbit. The red cross is the birth place. The open blue circle is the currant place. The cluster moves in Galactic center direction within Galactic plane, and as a result, it generally faces the impact of Galactic tidal forces.

For input, we used the cluster parameters presented: proper motions ($\mu _{\alpha } \cos \delta$, $\mu _{\delta }$), distance from the Sun, equatorial coordinates ($\alpha$, $\delta$), and radial velocity, which was calculated as an average from the Gaia DR3 data for member stars. Fig. 18 shows the integrated orbit of Czernik 38 in the Cartesian Galactocentric coordinate system, backward in time according to the cluster age determined in this study. The red cross indicates the birthplace of the cluster. According to the *z*-coordinate, the cluster oscillates about it every 92.54 million years, rising above the plane of the disk up to a maximum height of 181.76 pc. Therefore, Czernik 38 belongs to the very thin-disk component of the Galaxy. The apocenter $R_{apo}$ and the pericenter $R_{peri}$ are found to be 5.81 and 4.81 Kpc, respectively, which correspond to the eccentricity of the orbit ($e=( R_{apo}-R_{peri})/(R_{apo}+R_{peri})$) 0.095. The current coordinates $(x,\; y,\; z, r,\;\; v_x,\; v_y,\; v_z)$ are (5.08, 2.21,0.18, 5.55,−104.29, 186.48, −3.46), while the birthplace coordinates are (2.04, 5.25, −0.13, 5.63, −187.26, 92.66, 14.32). Moreover, the space velocity components U, V and W are 94, −47.61 and −10.49 km $s^{-1}$, respectively.

To summarize this section, the Czernik 38 cluster is located 5.55 kpc from the Galactic center and is moving parallel to the Galactic plane towards the Galactic center, following a nearly circular orbit.

**Table 4:** The orbital’s parameters.

No. of atars	Apocenter	Pericenter	Eccentricity	$R_{gal}$	$T_r$	vx	vy	vz	W	V	U	Ref.
	kpc	kpc	-	kpc	Gyr	km$s^{-1}$	km$s^{-1}$	km$s^{-1}$	km$s^{-1}$	km$s^{-1}$	km$s^{-1}$	
37	5.81	4.81	0.095	5.55	0.1	−104.29	186.48	−3.46	−20.85	−56.8	25.1	this work
1	8.02	6.19	0.129	6.67	-	91.46	241.64	2.8	-	-	-	^66^
1	-	-	-	-	-	-	-	2.60	−4.65	−13.66	82.72	^67^

## The Morphology and the Tidal Tail of Czernik 38

The evolution of an open cluster, influenced by internal or external forces, is reflected in its changes in shape. More than a century ago, the flattening of a moving cluster was postulated by^68^. An important aspect of OCs is their morphological structure, which is associated with features such as elongated shapes and tidal tails. The orientation of cluster elongation or tidal tail reflects the direction and nature of the gravitational tidal force.

### The elongation of Czernik 38

**Fig. 19:**
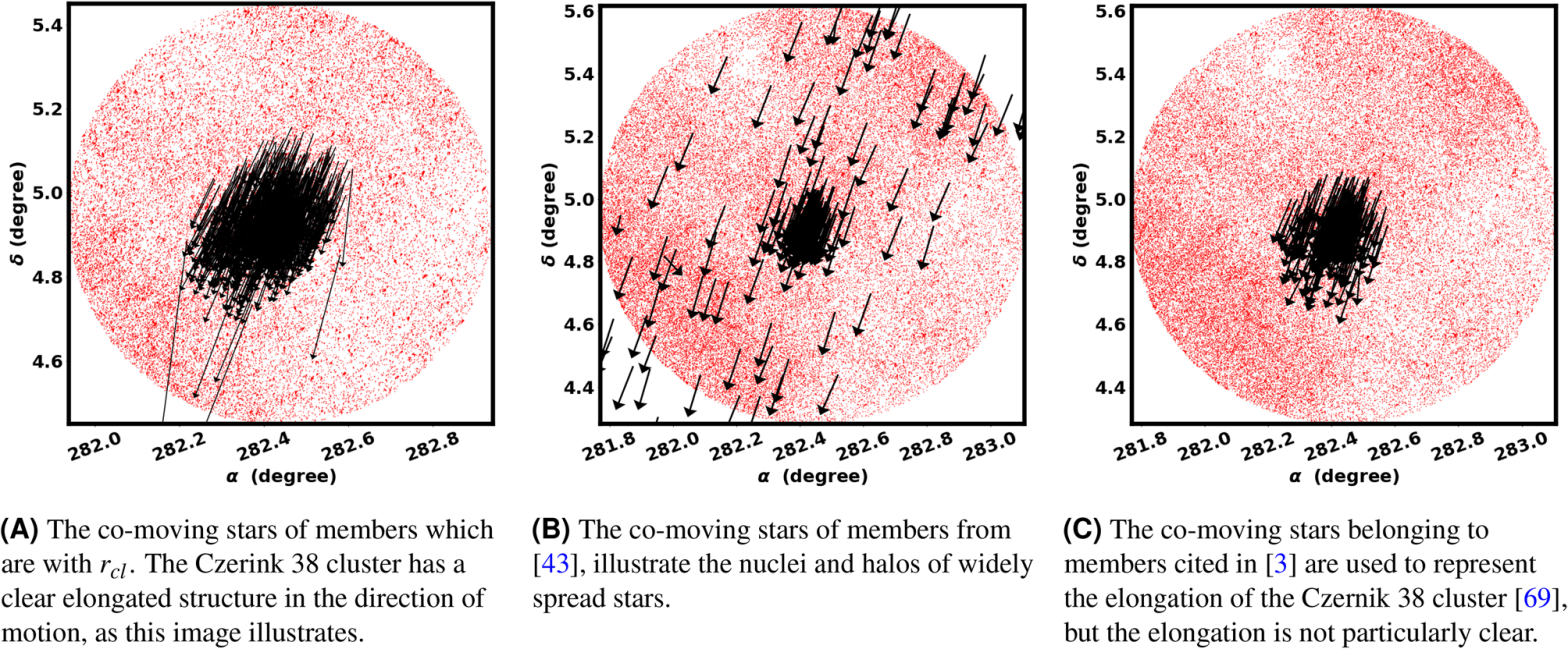
The co-moving star diagrams of Czernik 38, derived from Gaia DR3, are based on members from our research as well as other sources.

The analysis conducted by^43^ on 476 OCs utilized a probabilistic approach, revealing the elongated structure in all samples, with Czernik 38 being one of them. The co-moving stars associated with his members are illustrated in Fig. 19B. In addition, the RDP of these members is illustrated in Fig. 9^69^. conducted an analysis of the morphology of 1256 OCs employing nonparametric bivariate density estimation. They found the elongated shape of the majority of their samples, with Czernik 38 being one of them. The diagram of co-moving stars for their members is depicted in Fig. 19C. These strategies appear to experience some difficulties and commonly trend towards an elliptical shape in star clusters, along with an S-shaped configuration.

In this research, we have also found an elongated structure, as depicted in Fig. 19A, using a natural method that combines the HDBSCAN probability with the King model. Moreover, we identified the direction of elongation, which aligns with the direction of orbital motion. As indicated previously, this orientation is significant for elucidating the properties of the tidal force; please refer to subsection "The nature of the tidal force affected the Czernik 38" for more comprehensive information.

### The tidal tail of Czernik 38

**Fig. 20:**
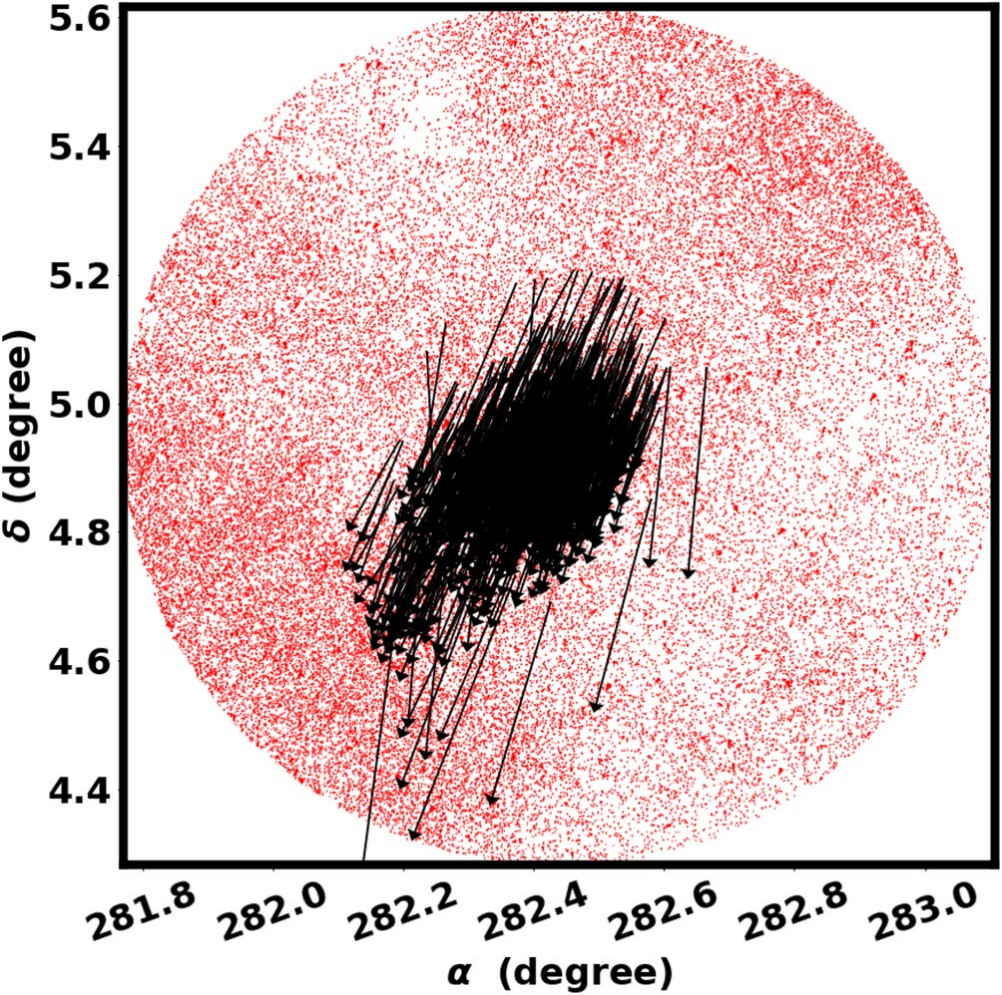
Our study reveals only the leading tidal tail of Czernik 38, with no trailing tails observed.

These tidal tails are stellar structures that extend from the main body of the cluster, arising from the gravitational interactions between the cluster and the Galactic potential or a giant molecular cloud^9^. Clusters that exhibit substantial elongation in their morphology tend to have tidal tails^17^. The pronounced elongation of Czernik 38 is the basis for our investigation into the tidal effects that extend beyond the radius of the cluster. Subsequently, we applied a probability of 80% outside the cluster radius, extending to 17 arcmin, up to the boundary of the new cluster. we have identified only a leading tidal tail (see Fig. 20) that is aligned with the orbital motion, directed towards the Galactic center; refer to Fig. 18. We agree with some results of^17^, where they identified extended structures in 46 OCs with elongated configurations, of which 20 clusters have tidal tails that are aligned with their orbital motions.

In our earlier investigation, we have also noted only a leading tidal tail that lies beyond the radius of the cluster, oriented in the direction of the orbital motion within the King 13 open cluster^25^. Conversely, some research utilizing 3D projection indicates that the tidal tail is made up of two distinct components: a leading tail and a trailing tail, which together produce an S-shaped configuration^15,70^. The issue may lie in their techniques, see section “Final note”.

### The nature of the tidal force affected the Czernik 38

Fundamentally, displacement is dynamically influenced by acceleration and the initial conditions of both position and velocity, rather than by the mass of the object. For instance, free fall towards the Earth, irrespective of air resistance, is independent of the mass of the object and is instead determined by gravitational acceleration. In other terms, objects of varying masses arrive at the Earth simultaneously.

In a similar manner, the stars located in the forefront of the cluster are less influenced by the gravitational forces exerted by the cluster. As a result, they undergo increased orbital acceleration relative to the rest of the cluster, which causes them to move more rapidly than the other stars present in the cluster. This dynamic scenario ultimately results in the creation of an elongated shape and possibly a leading tidal tail in the direction of cluster orbital motion, known as differential rotation tides. In addition, when considering tides that result from differential rotation, the part extending in front of the cluster will persist unchanged at the forefront throughout its orbital trajectory. Consequently, the influence of the differential rotation tide lasts for a long time. Therefore, we expect that this type of tide is more common.

In contrast, in the case of Galactic tides or those from any massive objects, the part of the cluster affected by the tide may change during its orbital journey. The orientation of the elongated structure or tail may not align with the direction of orbital motion. Consequently, it is imperative to recognize the direction of the tide, as it is essential to understand the nature of the tidal force.

In this work, we have fount the direction of the elongated structure and the tidal tail of Czernik 38 cluster in the direction of the orbital motion, as seen in Fig. 19A. Consequently, as we discussed earlier, this kind of tide is a result of differential rotation. However, this cluster is moving towards the Galactic center and may also be influenced by the Galactic tide.

## Final note

In Fig. 16, it is vital to stress that the distribution of distances is nearly Gaussian, and this is only associated with parallax errors, rather than the actual physical 3D distribution of stars or line of sight distance variation. In other words, any 3D distribution of stars or any density map based on coordinates will not be connected to the real physical position distribution of stars, resulting in a fake shape and false results.

In literature, the Astropy python package^71^ is used to calculate the Galactic Cartesian coordinates (X, Y, and Z) with right ascension, declination, and zero-point corrected parallaxes as input, see^72,73^ as example. The X, Y, and Z coordinates are associated with parallax errors and do not accurately reflect the line of sight distances of the individual member stars. Furthermore, zero point correction does not yield individual distances; it only adjusts the mean.

The stars located at the center of the Gaussian distribution signify the nucleus of the cluster, whereas the stars positioned at the peripheries of the Gaussian distribution denote the leading and trailing tails or elliptical shape, creating a deceptive shape.

## Summary and conclusions

Czerink 38 is a remarkably plentiful open star cluster, distinguished by its significant number of stars and unique features resulting from its specific position in the Galaxy. However, this cluster has not been extensively studied. Consequently, we conducted an in-depth analysis of the young open cluster Czernik 38, utilizing photometric and astrometric data from Gaia DR3, alongside 2MASS data for comparative purposes with Gaia’s color-magnitude diagram (CMD). The objective of our analysis was to improve the parameters of Czernik 38 in relation to the Gaia DR3 era, particularly concentrating on its kinematics, dynamics, morphology and structural aspects. Moreover, To estimate membership, we employed the pyUPMASK Python package along with the HDBSCAN algorithm. The key focus of this investigation is our method of evaluating membership probability based on the radius of each shell in the studied cluster, utilizing King model, rather than applying a single probability value to the entire cluster. The key results of our study are summarized as follows **:-**

1. In Czernik 38, we identified 938 $\pm$ 61 member stars with a total mass of 2769.7$\pm$ 59.50 $M_\odot$ inside radius 14.36$\pm$ 7.63 arcmin.

2) We found that the cluster’s distance modulus was 12.69$\pm$ 0.08 mag, which is equivalent to 3448$\pm$ 362 pc. We also found the color excess $E(G_{\text {BP}} - G_{\text {RP}})$ is 2.40 $\pm$ 0.04 mag. Our findings have been confirmed by a comparison of the Gaia CMD with 2MASS data, which offers an additional viewpoint on the photometric characteristics of the cluster. The 2MASS distance modulus and the color excess $E(J-Ks)$ are found to be 12.87 $\pm$ 0.93 mag (3749.73 $\pm$ 0.93 pc) and 0.89 $\pm$ 0.2 mag, respectively. We estimate the age of the cluster to be 115.0$\pm$ 20.3 Myr. One of the characteristics of this cluster is significant reddening, which is not uniformly distributed throughout the cluster.

3) The proper motion components ($\mu _{\alpha } \cos \delta$, $\mu _{\delta }$) and the parallax ($\varpi$) were measured as −2.41 $\pm$ 0.328 mas yr$^{-1}$, −5.263 $\pm$ 1.063 mas yr$^{-1}$ (tangential velocity is 93.28 $\pm$ 27.16 km $s^{-1}$), and 0.21 $\pm$ 0.083 mas, respectively. The mean distance derived from parallax measurements is approximately 3580.4 $\pm$ 230.5 pc, which is in excellent agreement with the photometric data from Gaia and 2MASS data, within the associated uncertainties.

4) We also identified 37 member stars with radial velocity data with average 46.1 $\pm$ 8.54 km $s^{-1}$, allowing us to compute the orbital parameters of Czernik 38 using the galpy Python package. The Czernik 38 moves in Galactic plane toward the Galactic center.

5) *One of our main results is that:* We identified a an elongated shape and a leading tidal tail aligned with the direction of orbital motion. This indicates that Chernik 38 is greatly affected by its orbital differential rotation, and this tide may be strengthened by the Galactic tide as it moves toward the Galactic center.

6) The coordinates, proper motions, and distances of individual stars in the majority of open clusters display Gaussian distributions. Such distributions result from errors, not from actual physical values. Thus, any 3D distribution of stars or any density map based on coordinates will not relate to the true physical position distribution of stars, resulting in an artificial shape and and misleading results

7) In general, we examined the area surrounding the cluster within a radius of 50 arcmin and employed a membership probability map in two dimensions $\alpha$ and $\delta$, with probability threshold of greater than 80.%. Two separate groups of stars were identified. The first group corresponds to the Czernik 38 cluster, whereas the second group represents a newly discovered cluster. The initial investigations indicate that it displays a King profile and a color-magnitude diagram (CMD) consistent with the isochrone of Czernik 38. This may indicate a binary cluster or a complex colliding system located within a densely populated area of stars and interstellar material. We plan to explore this intriguing subject further in our upcoming research, offering comprehensive insights into the features of this newly identified cluster.

8) We have identified a novel category of pre-main sequence stars that form a distinct branch in the right of Color-Magnitude Diagram (CMD). These stars exhibit lower temperatures and surface gravities compared to main sequence stars. This implies a considerable rate of star formation. The characteristic of this high star formation rate in this cluster corresponds with its relatively young age, significant reddening value, and its specific location amidst a concentration of stars and dense gases. In addition, we identified faint stars in both clusters, Czernik 38 and the new one, that exhibit bluer characteristics than main sequence stars, suggesting they could be white dwarf stars. These findings are intriguing because these stars are located in young clusters.

In summary, alongside the photometric analysis, we offer significant insights into the structure, stellar populations, stellar evolution, as well as the kinematics and dynamics of Czernik 38, emphasizing the potential of Gaia DR3 data for comprehensive research on open star clusters. Moreover, the accurate astrometry data derived from Gaia has significantly modified our understanding of this cluster and has led to the discovery of new phenomena, thanks to Gaia.

## Data Availability

Gaia DR3 and 2MASS data : are available for free in webpage https://vizier.cds.unistra.fr/

## References

[1] Lada, C. J. & Lada, E. A. Embedded Clusters in Molecular Clouds. *Annu. Rev. Astron. Astrophys* **41**, 57–115, 10.1146/annurev.astro.41.011802.094844 (2003). arXiv: astro-ph/0301540.

[2] Carraro, G., Geisler, D., Villanova, S., Frinchaboy, P. M. & Majewski, S. R. Old open clusters in the outer Galactic disk. *Astron. Astrophys.* **476**, 217–227. 10.1051/0004-6361:20078113 (2007).

[3] Cantat-Gaudin, T. & Anders, F. Clusters and mirages: cataloguing stellar aggregates in the Milky Way. *Astron. Astrophys.* **633**, A99. 10.1051/0004-6361/201936691 (2020).

[4] Madore, B. F., Freedman, W. L., Lee, A. J. & Owens, K. Milky Way Zero-point Calibration of the JAGB Method: Using Thermally Pulsing AGB Stars in Galactic Open Clusters. *Astrophys* **938**, 125. 10.3847/1538-4357/ac92fd (2022).

[5] Czernik, M. *New Open Star Clusters. Acta Astron.* **16**, 93 (1966).

[6] Dias, W. S., Alessi, B. S., Moitinho, A. & Lépine, J. R. D. New catalogue of optically visible open clusters and candidates. *Astron. Astrophys.* **389**, 871–873, 10.1051/0004-6361:20020668 (2002). arXiv: astro-ph/0203351.

[7] Maciejewski, G. Photometric Studies of Open Clusters: Be 95, Cze 21, Cze 38, Ju 11, King 17, and King 18. *Acta Astron.* **58**, 389 (2008).

[8] Carraro, G. & Seleznev, A. F. UBVI CCD photometry and star counts in nine inner disc Galactic star clusters. *Mon. Notices Royal Astron. Soc.* **419**, 3608–3623. 10.1111/j.1365-2966.2011.20010.x (2012).

[9] Küpper, A. H., Kroupa, P., Baumgardt, H. & Heggie, D. C. Tidal tails of star clusters. *Mon. Notices Royal Astron. Soc.* **401**, 105–120 (2010).

[10] Meingast, S., Alves, J. & Rottensteiner, A. Extended stellar systems in the solar neighborhood-v. discovery of coronae of nearby star clusters. *Astron. & Astrophys.* **645**, A84 (2021).

[11] Pang, X. et al. 3d morphology of open clusters in the solar neighborhood with gaia edr 3: its relation to cluster dynamics. *The Astrophys. J.* **912**, 162 (2021).

[12] Carrera, R. et al. Extended halo of ngc 2682 (m 67) from gaia dr2. *Astron. & Astrophys.* **627**, A119 (2019).

[13] Meingast, S. & Alves, J. Extended stellar systems in the solar neighborhood-i. the tidal tails of the hyades. *Astron. & Astrophys. ***621**, L3 (2019).

[14] Röser, S., Schilbach, E. & Goldman, B. Hyades tidal tails revealed by gaia dr2. *Astron. & Astrophys.* **621**, L2 (2019).

[15] Jerabkova, T. et al. The 800 pc long tidal tails of the hyades star cluster-possible discovery of candidate epicyclic overdensities from an open star cluster. *Astron. & Astrophys.* **647**, A137 (2021).

[16] Tarricq, Y. et al. Structural parameters of 389 local open clusters. *Astron. Astrophys.* **659**, A59. 10.1051/0004-6361/202142186 (2022).

[17] Bhattacharya, S., Rao, K. K., Agarwal, M., Balan, S. & Vaidya, K. A Gaia EDR3 search for tidal tails in disintegrating open clusters. *Mon. Notices Royal Astron. Soc.* **517**, 3525–3549. 10.1093/mnras/stac2906 (2022).

[18] Vaher, E., Hobbs, D., McMillan, P. & Prusti, T. Finding the dispersing siblings of young open clusters. Dynamical traceback simulations using Gaia DR3. *Astron. Astrophys.* **679**, A105, 10.1051/0004-6361/202346877 (2023).

[19] Boffin, H. M. J., Jerabkova, T., Beccari, G. & Wang, L. A tale of caution: the tails of NGC 752 are much longer than claimed. *Mon. Notices Royal Astron. Soc.* **514**, 3579–3592. 10.1093/mnras/stac1567 (2022).

[20] Ye, X., Zhao, J., Zhang, J., Yang, Y. & Zhao, G. Extended Tidal Tails of IC 4756 Detected by Gaia EDR3. *Astron. J.* **162**, 171. 10.3847/1538-3881/ac1f1f (2021).

[21] Yeh, F. C., Carraro, G., Montalto, M. & Seleznev, A. F. Ruprecht 147: A Paradigm of Dissolving Star Cluster. *Astron. J.* **157**, 115. 10.3847/1538-3881/aaff6c (2019).

[22] Gao, X. Discovery of Tidal Tails around the Old Open Cluster NGC 2506. *Astrophys* **894**, 48. 10.3847/1538-4357/ab8560 (2020).

[23] Pang, X. et al. 3D Morphology of Open Clusters in the Solar Neighborhood with Gaia EDR 3: Its Relation to Cluster Dynamics. *Astrophys* **912**, 162. 10.3847/1538-4357/abeaac (2021).

[24] Nikiforova, V. V., Kulesh, M. V., Seleznev, A. F. & Carraro, G. The Relation of the Alpha Persei Star Cluster with the Nearby Stellar Stream. *Astron. J.* **160**, 142. 10.3847/1538-3881/aba753 (2020).

[25] Ahmed, N. M. & Darwish, M. S. Tidal tail identification and detailed analysis of the open star cluster King 13 using Gaia DR3 and 2MASS. *Sci. Reports* **15**, 18033. 10.1038/s41598-025-96923-6 (2025). 10.1038/s41598-025-96923-6 PMC12102180 40410538

[26] Gaia Collaboration *et al.* Gaia Data Release 3. Summary of the content and survey properties. *Astron. Astrophys.* **674**, A1, 10.1051/0004-6361/202243940 (2023).

[27] Chambers, K. C. et al. The Pan-STARRS1 Surveys. *arXiv e-prints* arXiv:1612.05560, 10.48550/arXiv.1612.05560 (2016).

[28] Skrutskie, M. F. et al. The Two Micron All Sky Survey (2MASS). *Astron. J.* **131**, 1163–1183. 10.1086/498708 (2006).

[29] Bukowiecki, Ł., Maciejewski, G., Konorski, P. & Strobel, A. Open Clusters in 2MASS Photometry. I. Structural and Basic Astrophysical Parameters. *Acta Astron.* **61**, 231–246, 10.48550/arXiv.1107.5119 (2011).

[30] King, I. The structure of star clusters. I. an empirical density law. *Astron. J.* **67**, 471, 10.1086/108756 (1962).

[31] Bonatto, C. & Bica, E. The nature of the young and low-mass open clusters Pismis5, vdB80, NGC1931 and BDSB96. *Mon. Notices Royal Astron. Soc.* **397**, 1915–1925. 10.1111/j.1365-2966.2009.14877.x (2009).

[32] King, I. R. The structure of star clusters. III. Some simple dynamical models. *Astron. J.* **71**, 64, 10.1086/109857 (1966).

[33] Rangwal, G., Yadav, R. K. S., Durgapal, A., Bisht, D. & Nardiello, D. Astrometric and photometric study of NGC 6067, NGC 2506, and IC 4651 open clusters based on wide-field ground and Gaia DR2 data. *Mon. Notices Royal Astron. Soc.* **490**, 1383–1396. 10.1093/mnras/stz2642 (2019).

[34] Krone-Martins, A. & Moitinho, A. UPMASK: unsupervised photometric membership assignment in stellar clusters. *Astron. Astrophys.* **561**, A57. 10.1051/0004-6361/201321143 (2014).

[35] Pera, M. S., Perren, G. I., Moitinho, A., Navone, H. D. & Vazquez, R. A. pyUPMASK: an improved unsupervised clustering algorithm. *Astron. Astrophys.* **650**, A109. 10.1051/0004-6361/202040252 (2021).

[36] Pedregosa, F. et al. Scikit-learn: Machine Learning in Python. *J. Mach. Learn. Res.* **12**, 2825–2830, 10.48550/arXiv.1201.0490 (2011).

[37] Campello, R. J. G. B., Moulavi, D. & Sander, J. Density-based clustering based on hierarchical density estimates. In *Advances in Knowledge Discovery and Data Mining, 160–172 (Springer* (eds Pei, J. et al.) (Berlin Heidelberg, 2013).

[38] McInnes, L., Healy, J. & Astels, S. hdbscan: Hierarchical density based clustering. *The J. Open Source Softw.* **2**, 205, 10.21105/joss.00205 (2017).

[39] Zhong, J., Chen, L., Jiang, Y., Qin, S. & Hou, J. New Insights into the Structure of Open Clusters in the Gaia Era. *Astron. J.* **164**, 54. 10.3847/1538-3881/ac77fa (2022).

[40] Gao, X. 5D memberships and fundamental properties of the old open cluster NGC 6791 based on Gaia-DR2. *Astrophys. Space Sci.* **365**, 24. 10.1007/s10509-020-3738-2 (2020).

[41] van Groeningen, M. G. J., Castro-Ginard, A., Brown, A. G. A., Casamiquela, L. & Jordi, C. A machine-learning-based tool for open cluster membership determination in Gaia DR3. *Astron. Astrophys.* **675**, A68. 10.1051/0004-6361/202345952 (2023).

[42] Hunt, E. L. & Reffert, S. Improving the open cluster census-iii. using cluster masses, radii, and dynamics to create a cleaned open cluster catalogue. *Astronomy & Astrophysics* **686**, A42 (2024).

[43] Kos, J. Tidal tails of open clusters. *Astron. Astrophys.* **691**, A28. 10.1051/0004-6361/202449828 (2024).

[44] Marigo, P. et al. A New Generation of PARSEC-COLIBRI Stellar Isochrones Including the TP-AGB Phase. *Astrophys. J.* **835**, 77. 10.3847/1538-4357/835/1/77 (2017).

[45] Spada, F., Demarque, P., Kim, Y. C., Boyajian, T. S. & Brewer, J. M. The Yale-Potsdam Stellar Isochrones.* Astrophys. J.* **838**, 161. 10.3847/1538-4357/aa661d (2017).

[46] Bressan, A. et al. PARSEC: stellar tracks and isochrones with the PAdova and TRieste Stellar Evolution Code. *Mon. Notices Royal Astron. Soc.* **427**, 127–145. 10.1111/j.1365-2966.2012.21948.x (2012).

[47] Ahmed, N. M. & Tadross, A. L. The photometry and kinematics studies of NGC 2509 derived from Gaia DR3. *Sci. Reports* **15**, 17676. 10.1038/s41598-025-00383-x (2025). 10.1038/s41598-025-00383-x PMC12095638 40399287

[48] Wang, S. & Chen, X. The Optical to Mid-infrared Extinction Law Based on the APOGEE, Gaia DR2, Pan-STARRS1, SDSS, APASS, 2MASS, and WISE Surveys. *Astrophys. J.* **877**, 116. 10.3847/1538-4357/ab1c61 (2019).

[49] Cardelli, J. A., Clayton, G. C. & Mathis, J. S. The Relationship between Infrared, Optical, and Ultraviolet Extinction. *Astrophys. J.* **345**, 245. 10.1086/167900 (1989).

[50] O’Donnell, J. E. R v-dependent Optical and Near-Ultraviolet Extinction. *Astrophys* **422**, 158. 10.1086/173713 (1994).

[51] Abdurro’uf, et al. The Seventeenth Data Release of the Sloan Digital Sky Surveys: Complete Release of MaNGA, MaStar, and APOGEE-2 Data. *Journal, Suppl* **259**, 35. 10.3847/1538-4365/ac4414 (2022).

[52] Ahmed, N. M., Bendary, R., Samir, R. M. & Elhosseiny, E. G. A deep investigation of the poorly studied open cluster King 18 using CCD VRI, Gaia DR3 and 2MASS. *Sci. Reports* **14**, 23777. 10.1038/s41598-024-70133-y (2024). 10.1038/s41598-024-70133-y PMC11467434 39389984

[53] Anders, F. et al. Photo-astrometric distances, extinctions, and astrophysical parameters for Gaia EDR3 stars brighter than G = 18.5. *Astron. Astrophys.* **658**, A91, 10.1051/0004-6361/202142369 (2022).

[54] Richer, H. B. et al. Massive White Dwarfs in Young Star Clusters. *Astrophys. J.* **912**, 165. 10.3847/1538-4357/abdeb7 (2021).

[55] Dias, W. S. et al. Updated parameters of 1743 open clusters based on Gaia DR2. *Mon. Notices Royal Astron. Soc.* **504**, 356–371. 10.1093/mnras/stab770 (2021).

[56] Cantat-Gaudin, T. et al. Painting a portrait of the Galactic disc with its stellar clusters. *Astron. Astrophys.* **640**, A1. 10.1051/0004-6361/202038192 (2020).

[57] Lindegren, L. et al. Gaia Early Data Release 3. Parallax bias versus magnitude, colour, and position. *Astron. Astrophys.* **649**, A4, 10.1051/0004-6361/202039653 (2021).

[58] Bailer-Jones, C. A. L., Rybizki, J., Fouesneau, M., Demleitner, M. & Andrae, R. Estimating Distances from Parallaxes. V. Geometric and Photogeometric Distances to 1.47 Billion Stars in Gaia Early Data Release 3. *Astron. J.* **161**, 147, 10.3847/1538-3881/abd806 (2021).

[59] Poovelil, V. J. et al. Open Cluster Chemical Homogeneity throughout the Milky Way. *Astrophys. J.* **903**, 55. 10.3847/1538-4357/abb93e (2020).

[60] Sartoretti, P. et al. Gaia Data Release 2. *Processing the spectroscopic data. Astron. Astrophys.* **616**, A6. 10.1051/0004-6361/201832836 (2018).

[61] Cropper, M. et al. Gaia Data Release 2. *Gaia Radial Velocity Spectrometer. Astron. Astrophys.* **616**, A5. 10.1051/0004-6361/201832763 (2018).

[62] Antoja, T. et al. A dynamically young and perturbed Milky Way disk. *Nature* **561**, 360–362. 10.1038/s41586-018-0510-7 (2018). 30232428 10.1038/s41586-018-0510-7

[63] Bovy, J. galpy: A python Library for Galactic Dynamics. *Journal, Suppl* **216**, 29. 10.1088/0067-0049/216/2/29 (2015).

[64] Miyamoto, M. & Nagai, R. Three-dimensional models for the distribution of mass in galaxies. *PASJ* **27**, 533–543 (1975).

[65] Navarro, J. F., Frenk, C. S. & White, S. D. M. Simulations of X-ray clusters. *Mon. Notices Royal Astron. Soc.* **275**, 720–740, 10.1093/mnras/275.3.720 (1995). arXiv: astro-ph/9408069.

[66] Tarricq, Y. et al. 3D kinematics and age distribution of the open cluster population. *Astron. Astrophys.* **647**, A19. 10.1051/0004-6361/202039388 (2021).

[67] Soubiran, C. et al. Open cluster kinematics with Gaia DR2. *Astron. Astrophys.* **619**, A155. 10.1051/0004-6361/201834020 (2018).

[68] Jeans, J. H. On the law of distribution in star-clusters. *Mon. Notices Royal Astron. Soc.* **76**, 567. 10.1093/mnras/76.7.567 (1916).

[69] Hu, Q., Zhang, Y. & Esamdin, A. Decoding the morphological evolution of open clusters. *Astron. Astrophys.* **656**, A49. 10.1051/0004-6361/202141460 (2021).

[70] Dinnbier, F. & Kroupa, P. Tidal tails of open star clusters as probes of early gas expulsion-i. a semi-analytic model. *Astron.& Astrophys.* **640**, A84 (2020).

[71] Astropy Collaboration et al. The Astropy Project: Sustaining and Growing a Community-oriented Open-source Project and the Latest Major Release (v5.0) of the Core Package. *Astrophys. J.* **935**, 167, 10.3847/1538-4357/ac7c74 (2022).

[72] Xu, M. et al. Nearby open clusters with tidal features: Golden sample selection and 3D structure. *Astron. Astrophys.* **698**, A156. 10.1051/0004-6361/202554212 (2025).

[73] Hu, Q., Qin, S., Luo, Y. & Li, Y. 3D projection analysis: Characterizing the morphological stability of nearby open clusters. *Astron. Astrophys.* **693**, A125. 10.1051/0004-6361/202451243 (2025).

